# Diabetes comorbidities in low- and middle-income countries: An umbrella review

**DOI:** 10.7189/jogh.11.04040

**Published:** 2021-07-24

**Authors:** Anastasia A Lam, Alexander Lepe, Sarah H Wild, Caroline Jackson

**Affiliations:** 1 Usher Institute, University of Edinburgh, Edinburgh, UK; 2 School of Geography and Sustainable Development, University of St Andrews, St Andrews, UK; 3 Max Planck Institute for Demographic Research, Rostock, Germany; 4 Department of Health Sciences, University Medical Center Groningen, University of Groningen, Groningen, the Netherlands

## Abstract

**Background:**

Diabetes mellitus, particularly type 2 diabetes, is a major public health burden globally. Diabetes is known to be associated with several comorbidities in high-income countries. However, our understanding of these associations in low- and middle-income countries (LMICs), where the epidemiological transition is leading to a growing dual burden of non-communicable and communicable disease, is less clear. We therefore conducted an umbrella review to systematically identify, appraise and synthesise reviews reporting the association between diabetes and multiple key comorbidities in LMICs.

**Methods:**

We searched Medline, Embase, Global Health, and Global Index Medicus from inception to 14 November 2020 for systematic reviews, with or without meta-analyses, of cohort, case-control or cross-sectional studies investigating the associations between diabetes and cardiovascular disease (CVD), chronic kidney disease (CKD), depression, dengue, pneumonia, and tuberculosis within LMICs. We sought reviews of studies focused on LMICs, but also included reviews with a mixture of high-income and at least two LMIC studies, extracting data from LMIC studies only. We conducted quality assessment of identified reviews using an adapted AMSTAR 2 checklist. Where appropriate, we re-ran meta-analyses to pool LMIC study estimates and conduct subgroup analyses.

**Results:**

From 11 001 articles, we identified 14 systematic reviews on the association between diabetes and CVD, CKD, depression, or tuberculosis. We did not identify any eligible systematic reviews on diabetes and pneumonia or dengue. We included 269 studies from 29 LMICs representing over 3 943 083 participants. Diabetes was positively associated with all comorbidities, with tuberculosis having the most robust evidence (16 of 26 cohort studies identified in total) and depression being the most studied (186 of 269 studies). The majority (81%) of studies included were cross-sectional. Heterogeneity was substantial for almost all secondary meta-analyses conducted, and there were too few studies for many subgroup analyses.

**Conclusions:**

Diabetes has been shown to be associated with several comorbidities in LMICs, but the nature of the associations is uncertain because of the large proportion of cross-sectional study designs. This demonstrates the need to conduct further primary research in LMICs, to improve, and address current gaps in, our understanding of diabetes comorbidities and complications in LMICs.

Diabetes affected an estimated 463 million people in 2019, a number that is expected to grow to approximately 700 million by 2045 [1]. Of people with diabetes, about 90% have type 2 diabetes and almost 80% live in low- and middle-income countries (LMICs), where prevalence is increasing most rapidly [1]. This increasing prevalence reflects the epidemiological transition, with rapid urbanisation leading to harmful changes in diet and lifestyle behaviours [2]. As more people develop diabetes in LMICs which already have a major burden of infectious diseases, health systems must adapt to provide the joint care necessary to treat and manage the comorbidities with which diabetes is associated. However, for this to be done it is important to understand which conditions related to diabetes are most relevant in LMICs and establish the strength of evidence for the association of diabetes with these conditions. Although the focus of this paper is on type 2 diabetes, we will use the general term diabetes as many studies do not distinguish between type 1 or type 2 diabetes. This review uses the term comorbidities, rather than comorbidities and complications, of diabetes, but we acknowledge that certain conditions such as macrovascular disease or depression may exist as either a comorbidity or as a complication of diabetes.

Much of the evidence on diabetes comorbidities stems from high-income countries (HICs) [3-6]. However, these findings cannot necessarily be extrapolated to LMICs, where context-specific factors might influence associations. Moreover, various infectious diseases which may be related to diabetes are more common in LMICs. We identified six key conditions for which diabetes is thought to be a risk factor or for which there may be a bi-directional association, and which are particularly relevant to the LMIC setting. Non-communicable diseases including cardiovascular disease (CVD), chronic kidney disease (CKD), and depression are highly prevalent in HICs and associated with diabetes, but are also leading contributors to the burden of disease as measured by disability-adjusted life years (DALYs) in LMICs [7-11]. Three communicable diseases which have been linked to diabetes are dengue and tuberculosis (TB), which are most prevalent in LMICs, and pneumonia which is common globally [12,13].

Given the expanding literature on the association between diabetes and comorbidities, there is a need to synthesise this information to give a comprehensive overview of this topic in the context of LMIC settings. We therefore conducted an umbrella review of systematic reviews and meta-analyses to provide an overview of the breadth and strength of the existing evidence on the association between diabetes and the aforementioned comorbidities in LMICs.

## METHODS

This umbrella review was performed according to the guidelines laid out by Aromataris et al. [14].

### Search strategy

We searched Medline, Embase, Global Health, and Global Index Medicus from inception up to 14 November 2020 to identify systematic reviews of observational studies on the association between diabetes and CVD, CKD, dengue, depression, pneumonia, and TB. CVD encompassed coronary heart disease, myocardial infarction, angina, stroke and transient ischaemic attack, since diabetes has been shown to be associated with a markedly higher risk of these conditions [15]. The search included variations of the following as MeSH terms or keywords: diabetes, CVD, CKD, dengue, depression, pneumonia, and TB, as well as terms for LMICs, including individual country names (Appendix S1 in the **Online Supplementary Document**) [16]. We also perused reference lists of identified relevant studies. Two authors (AAL and AL) independently screened titles, abstracts, and full-text articles. Any discrepancies were resolved through discussion.

### Inclusion and exclusion criteria

We included systematic reviews, with or without meta-analyses, if they included cross-sectional, case-control or cohort primary studies. Primary studies had to have evaluated the prevalence or incidence of the relevant comorbidity in people with diabetes or the role of diabetes as a risk factor for the subsequent development of the condition. We included reviews of primary studies conducted solely in LMICs, or from a mixture of HICs and LMICs if at least two studies within a review were from an LMIC. Only LMIC studies from the latter were used in the analysis.

We excluded reviews which: focused on the association between diabetes and its comorbidities in the context of a third disease (for example, those which solely examined the association between diabetes and TB in an HIV-positive population); included diabetes as the outcome or focused solely on type 1 diabetes mellitus or gestational diabetes; examined studies of mortality outcomes; or were not conducted systematically (ie, narrative reviews, commentaries, editorials). Although our search strategy did not exclude non-English articles, we ultimately excluded articles written in languages other than English, Spanish, or Portuguese.

### Study selection

If multiple reviews included overlapping primary studies, the review which was most comprehensive (eg, greater number of LMIC studies) was included. If reviews on the same outcome focused on different settings (for example, one country vs multiple), both were included. If there were prevalence and incidence or association reviews on the same comorbidity, provided they did not overlap in terms of primary studies or settings, all eligible studies were included.

### Data extraction

Data were extracted independently by two authors (AAL and AL) using a standardised form adapted from the Joanna Briggs Institute [14]. Any discrepancies were resolved through discussion. If a review included primary studies from both HICs and LMICs, we only extracted data pertaining to LMIC studies. We categorised LMICs based on the World Bank’s historical income classification at the beginning of the primary study period [17]. Thus, the same country may be classified in different income levels depending on when the study began. We extracted the following data from reviews: first author; year; number of included LMIC primary studies; study designs; study settings; number of participants; country of primary study; population; exposure; exposure ascertainment; comparators (if any); outcome; outcome ascertainment; databases searched; and quality assessment method. For eligible meta-analyses, we extracted the following additional data items: study period; meta-analysis metric; number of outcomes (if more than one); type of model; factors adjusted for; effect estimate with 95% confidence interval; variables that were adjusted for; measures of heterogeneity (Q and/or I^2^) with *P* values; and measures of publication bias (Egger’s Test and/or Begg’s Test). If necessary data were not provided, or if there were any discrepancies identified in the review, we examined primary studies to extract the appropriate information.

### Quality appraisal

The methodological quality of both systematic reviews and meta-analyses was assessed independently by two authors (AAL and AL) using an adapted version of the AMSTAR 2 checklist (Appendix S2 in the **Online Supplementary Document**) [18]. AMSTAR 2 is an improved version of AMSTAR, a tool designed for the critical appraisal of systematic reviews of non-randomised and/or randomised studies of health care interventions. AMSTAR 2 is more inclusive to systematic reviews of observational studies and is comprised of the following domains: methods (ie, proper reporting of PICO, protocol, search strategy, eligibility criteria, data extraction and analysis), quality assessment (ie, risk of bias appraisal), and meta-analysis methods (ie, appropriate methods, investigation of heterogeneity and publication bias). Where the AMSTAR 2 checklist asked about randomised and non-randomised studies separately, we removed the parts related to randomised studies so the focus could be on observational studies. We also included an additional question on the type of quality appraisal tool used and whether quality descriptions were provided.

The checklist uses a rating system of high, moderate, low, and critically low confidence in the results, which is based on how well the identified review covers the domains deemed to be most important to the validity of umbrella reviews [18]. The critical domains included: adequate search strategy, sufficient reporting of study characteristics, risk of bias/quality appraisal, appropriate meta-analytical methods, accounting for risk of bias during results interpretation, and assessment of publication bias. If one critical domain was missing, the review was automatically dropped to a low confidence. If two or more critical domains were missing, the review was given a critically low confidence and thus excluded.

### Statistical analysis

For systematic reviews which did not conduct a meta-analysis, or for which we did not conduct a secondary meta-analysis, we conducted narrative syntheses, which comprised a summary of primary study characteristics and main findings. For reviews which did include a meta-analysis, all included meta-analyses were re-run using random-effect models and summary estimates were pooled based on the reported effect estimates from each review [19]. Where reviews included both HIC and LMIC studies in their meta-analysis, we only included the LMIC studies in our secondary meta-analysis. We extracted the following information for each primary study from an LMIC to conduct the secondary meta-analysis: first author; year; country of study; study design; study size; income classification of country at first year of study period; study population; any information on diagnosis or type of exposure or outcome; effect estimate; and 95% confidence intervals (CI).

We assessed heterogeneity using the *I^2^* metric, with *I^2^*<50%, *I^2^* = 50%-75% and *I^2^*>75% suggesting low, moderate, and substantial heterogeneity, respectively [20]. If substantial heterogeneity was present and there were a sufficient number of studies, pre-defined subgroup analyses (study design and country income level) were conducted to explore potential sources of heterogeneity. If the identified meta-analysis conducted relevant subgroup analyses, we also re-ran those analyses if included primary studies differed or if the original analysis was not pooled using random effects models. If a review included at least ten primary studies, we explored publication bias using a funnel plot and Egger’s test to evaluate whether smaller studies contribute more to the effect estimate compared with larger studies [21]. For prevalence meta-analyses, we did not assess publication bias because these funnel plots were found to be inaccurate [22].

We used Stata version 16 (StataCorp LLC, College Station, Texas, USA) to conduct secondary meta-analyses [23].

## RESULTS

**Figure 1:**
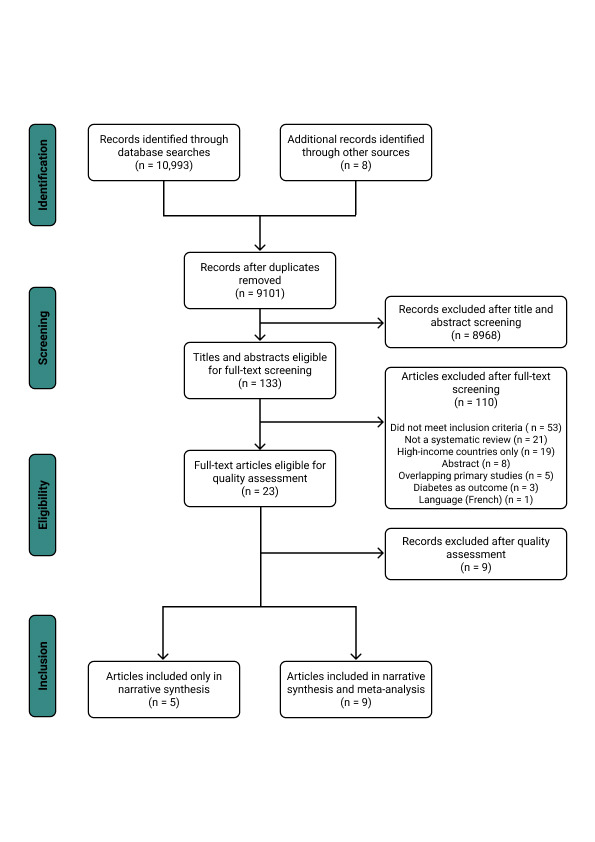
PRISMA flow diagram detailing the study selection process.

Our search identified 11 001 articles, of which 23 were eligible for inclusion following full-text screening (**Figure 1**). Reviews which were excluded following full-text screening are listed in Table S1 in the **Online Supplementary Document**. After quality assessment, we excluded nine reviews of critically low confidence mainly due to insufficient primary study details and lack of risk of bias or quality appraisal (Table S2 in the **Online Supplementary Document**). We therefore included a total of 14 reviews, which focused on diabetes in relation to CVD (two reviews) [24,25], CKD (two reviews) [26,27], depression (five reviews) [28-32], and TB (five reviews) [33-37] (**Table 1**). We did not identify any eligible reviews on the association between diabetes and pneumonia. For dengue, one review met our inclusion criteria [38], but was rated as being of critically low confidence and was thus excluded from our final selection (Table S2 in the **Online Supplementary Document**). Therefore, we included five reviews in the narrative synthesis and nine in the secondary meta-analysis.

**Table 1:** Distribution of primary study designs and number of participants by comorbidity and country income level from included systematic reviews and meta-analyses

	Low- and middle-income countries					High-income countries*						
	**Cohort**	**Case-control**	**Cross-sectional**	**Primary studies**	**Participants**	**Cohort**	**Case-control**	**Cross-sectional**	**Primary studies**	**Participants**	**% LMIC primary studies**	**% LMIC participants**
**Depression**	0	3	183	186	528 987†	8	7	1	16	838 399	86.1	38.2
**Chronic kidney disease**	4	1	9	14	5 189	69	0	0	69	820 138	2.82	0.14
**Cardiovascular disease^‡^**	6	6	10	22	3 108 248	20	1	21	42	1 440 950	34.4	68.3
**Tuberculosis**	16	15	17	47^§^	300 659	19	15	26	53	58 286 930	47	0.51
**TOTAL** **(%)**	**26 (9.67%)**	**25 (9.29%)**	**219 (81.4%)**	**269**	**3** **943** **083**	**116 (64.4%)**	**23 (12.8%)**	**48 (26.7%)**	**180**	**61** **386** **417**	**59.9**	**6.42**

LMIC – low- and middle-income country

*These primary studies are from the systematic reviews and meta-analyses which included studies from high-income countries and were included in this umbrella review.

†This number does not include the participants from Uphoff et al. [29] since they did not provide individual study characteristics.

‡One primary study from Einarson et al. [24] included low, middle, and high-income countries and was thus not included in this table.

§One primary study from McMurry et al. [34] included data on two separate cross-sectional studies, so both were included in the cross-sectional but not primary study count.

This umbrella review represented data from over 3 943 083 participants across 26 cohort, 25 case-control, and 219 cross-sectional studies from 29 LMICs. This number of participants does not include those from the 41 cross-sectional studies included in the included review by Uphoff et al. [29] which did not provide this information. Of the 14 included reviews, only eight [25,27-31,33,34] focused solely on LMICs. The other six reviews included a mix of primary studies from HICs and LMICs, with the majority often being from HICs. As shown in **Table 2**, there were relatively few cohort studies, and among the mixed income level reviews, there were far fewer cohort studies conducted in LMICs than in HICs. Among the diabetes comorbidities of interest, cohort studies were used most often to investigate TB, with 16 cohort studies identified. Depression was the most studied comorbidity in terms of volume of primary studies, although almost all were cross-sectional (183 of 186 studies).

**Table 2:** Characteristics of included systematic reviews and meta-analyses, which reported data on relevant diabetes comorbidities from at least two LMIC countries

Outcome	Study (Design) [LMIC or LMIC/HIC]	LMIC primary studies	Countries included (n)	LMIC primary study designs	No. LMIC participants	Aim of review	Population, Exposure, Comparison*, Outcome	Databases searched	Quality assessment in review	AMSTAR 2 quality rating
CVD	Einarson, 2018 [24] (Systematic review) [LMIC/HIC]	14	Brazil (2), Cameroon (1), China (5), India (2), Indonesia (1), Iraq (1), Russia (1), Thailand (1)	Cohort (6)	3 105 760	Estimate current prevalence of CVD amongst adults with type 2 diabetes between 2007-2017 across the world	*Population*: Adults with type 2 diabetes	Medline, Embase, PubMed, conference presentations/abstracts	STROBE	Moderate
				CS (8)			*Exposure*: Type 2 diabetes			
							*Outcome*: CVD prevalence			
CVD	Poorzand, 2019 [25] (Meta-analysis) [LMIC]	8	Iran	CC (6)	2488	Understand the association between diabetes and premature coronary artery disease	*Population*: Iranian patients with premature coronary artery disease	Web of Science, PubMed, Embase, Scientific Information Database	Joanna Briggs Institute Critical Appraisal Checklist	Low
				CS (2)			*Exposure*: Diabetes			
							*Outcome*: Premature coronary artery disease			
CKD	Koye, 2017 [26] (Systematic review) [LMIC/HIC]	2	Iran (1), Thailand (1)	Cohort (2)	1114	Synthesise information on the incidence of CKD in people with diabetes, in order to inform policy and prevention options.	*Population*: People with diabetes	Medline, Embase, CINAHL	Newcastle-Ottawa Scale	Moderate
							*Exposure*: Diabetes			
							*Outcome*: CKD incidence			
CKD	Shiferaw, 2020 [27] (Meta-analysis) [LMIC]	12	Ethiopia	Cohort (2)	4075	Estimate the prevalence of CKD and associated factors amongst patients with diabetes in Ethiopia	*Population*: People with diabetes	PubMed, Scopus, Google Scholar, African Journals Online, Wily Online Library	Newcastle-Ottawa Scale	Low
				CC (1)			*Exposure*: Diabetes			
				CS (9)			*Outcome*: CKD			
Depression	Mendenhall, 2014 [28] (Systematic review) [LMIC]	48	Bangladesh (3), Brazil (5), China (5), Egypt (1), India (8), Iran (4), Iraq (1), Jordan (1), Mexico (8), Nigeria (4), Oman (1), Pakistan (3), Russia (2) South Africa (1), Turkey (1)	CS	12 511	Determine the current global situation of comorbid diabetes and depression.	*Population*: Adults with comorbid type 2 diabetes and depression	Medline, PubMed, PsychInfo	None	Low
							*Exposure*: Type 2 diabetes			
							*Outcome*: Depression			
Depression	Uphoff, 2019 [29] (Systematic review) [LMIC]	41; 43 estimates	Bangladesh, India, Pakistan	CS	-	Estimate the prevalence of common mental disorders in adults with non-communicable diseases in Bangladesh, India, and Pakistan	*Population*: Adults with non-communicable diseases, including diabetes	BRAC Research & Publication website, Cochrane Database of Systematic Reviews (Wiley), Database of Abstracts of Reviews of Effect (Wiley), Global Health (Ovid), Global Index Medicus (WHO), Health Technology Assessment Database (Wiley), IndMed (ICMR-NIC), Ovid MEDLINE(R), PakMediNet (PakCyber), PsycINFO (Ovid), World Bank Group Research and Publications: Documents and Reports website	AXIS tool	Moderate
							*Exposure*: Diabetes			
							*Outcome*: Depression			
Depression	Hussain, 2018 [30] (Meta-analysis) [LMIC]	43	India	CS	10 270	Estimate the prevalence of depression amongst people with type 2 diabetes	*Population*: People with type 2 diabetes in India	Medline, Embase	Newcastle-Ottawa Scale	Moderate
							*Exposure*: Type 2 diabetes			
							*Outcome*: Depression			
Depression	Khalighi, 2019 [31] (Meta-analysis) [LMIC]	44	Iran	CS	10 349	Determine the prevalence of depression and anxiety in patients with diabetes in Iran	*Population:* Patients with diabetes in Iran	Iranian Journal Database, Barakat Knowledge Network System, Scientific Information Database, Iranian National Library, Regional Information Center for Science and Technology, Iranian Research Institute for Information Science and Technology, PubMed/Medline, Science Direct, Embase, Scopus, Cochrane Library, Web of Science, Google Scholar	Newcastle-Ottawa Scale	Low
							*Exposure:* Diabetes			
							*Outcome:* Prevalence of depression and anxiety			
Depression	Wang, 2019 [32] (Meta-analysis) [LMIC/HIC]	10	Brazil (1), China (1), India (3), Iran (2), Iraq (1), Nigeria (1), Uganda (1)	CC (3)	495 857	Determine the prevalence of major depressive disorder in people with type 2 diabetes	*Population:* Patients with type 2 diabetes	PubMed, Embase, PsycINFO, Cochrane, reference lists	“Guidelines for evaluating prevalence studies” Boyle, 1998	Moderate
							*Exposure:* Type 2 diabetes			
				CS (7)			*Comparison:* People without type 2 diabetes			
							*Outcome:* Prevalence/OR of major depressive disorder			
TB	Bailey and Ayles, 2017 [33] (Systematic review) [LMIC]	3	Guinea-Bissau (1), Tanzania (2)	CC	2870	Determine current evidence for the association between the prevalence of type 2 diabetes and the incidence or prevalence of TB in Africa and how HIV may modify the association.	*Population*: African population	Embase, Global Health, PubMed	Assessed by ascertaining study definitions for exposure variable, outcome variable, and comparison group and determining the variables adjusted for in the analysis - no standardised tool used	Moderate
							*Exposure*: Type 2 diabetes			
							*Comparison:* HIV/non-HIV			
							*Outcome*: Active pulmonary TB			
TB	McMurry, 2018 [34] (Systematic review) [LMIC]	17^†^	Bangladesh (1), China (3), Ethiopia (2), India (3), Iran (1), Marshall Islands (1), Micronesia (1), Pakistan (1), South Africa (3), Tanzania (1), Zambia (1)	PC (5)	60 678	Summarise information on current research findings regarding the co-prevalence of TB and diabetes in LMICs	*Population*: People with diabetes	PubMed, Embase, Medline, PsychINFO	Structured quality assessment scoring system adopted from a previous review	Moderate
				RC (1)			*Exposure*: Diabetes (excluding studies that solely focused on type 1 diabetes)			
				CC (1)			*Outcome*: TB			
				CS (11)						
TB	Al-Rifai, 2017 [35] (Meta-analysis) [LMIC/HIC]	10	China (2), Croatia (1), India (1), Indonesia (1), Mexico (1), Republic of Kiribati (1), Russia (1), Tanzania (1), Thailand (1)	Cohort (4)	223 887	Review literature on the association between diabetes and TB and determine the strength of the association.	*Population*: Adults	Medline, Embase	Cochrane Guidelines for Systematic Reviews	Moderate
				CC (5)			*Exposure*: Diabetes			
				CS (1)			*Outcome*: Active TB			
LTB	Lee, 2017 [36] (Meta-analysis) [LMIC/HIC]	2	China (1), Thailand (1)	CS	3755	Investigate the association between diabetes and LTB to inform screening programmes.	*Population*: Adults	PubMed, Embase	Newcastle-Ottawa Scale	Moderate
							*Exposure*: Diabetes			
							*Outcome*: LTB			
MDR-TB	Tegegne, 2018 [37] (Meta-analysis) [LMIC/HIC]	15^‡^	Bangladesh (1), China (2), Egypt (1), Georgia (2), Indonesia (1), Iran (1), Mexico (4), Peru (1), Thailand (1), Turkey (1)	PC (5)	9469	Determine the association between diabetes and MDR-TB	*Population*: TB patients	PubMed, Embase, Web of Science, WHO Global Health Library	Newcastle-Ottawa Scale (Case-control and cohort) and Agency for Healthcare Research and Quality tool (Cross-sectional)	High
				RC (1)			*Exposure*: Diabetes			
				CC (6)			*Comparison:* People without diabetes			
				CS (3)			*Outcome*: MDR-TB			

CC – case-control, CKD – chronic kidney disease, CS – cross-sectional, CVD – cardiovascular disease, HIC – high income country, LMIC – low- and middle-income country, LTB – latent tuberculosis, MDR-TB – multi-drug resistant tuberculosis, PC – prospective cohort, RC – retrospective cohort, TB – tuberculosis

*The ‘Comparison’ group is only applicable to case-control studies.

†17 papers, but one paper covered two cross-sectional studies from two different countries, hence both were counted in the study design and country tallies.

‡15 primary studies, but two studies each had two separate analyses in previously vs newly diagnosed TB patients, thus 17 estimates were included in the meta-analysis.

It is difficult to provide exact numbers of primary studies per country income level since assignment of studies to income level varied depending on the beginning of the study period. Nevertheless, the majority of LMIC primary studies were from a middle-income country. All WHO regions were represented, with eight countries from the African Region, six from the Eastern Mediterranean Region, four from each of the European, South-East Asia, and Western Pacific Regions, and three from the Region of the Americas. Iran and India had the most studies at 61 and 60, respectively, followed by China (19), Ethiopia (14), and Mexico (13). Eleven countries were only represented once, and the remaining countries had generally 5 or fewer studies. Except for CKD, each outcome had at least one study from each WHO region, with TB including the greatest number of countries (22). Seventy-eight percent of participants (3 060 517) came from a single Russian cross-sectional study [39] and many studies were from Iran and India due to country-specific reviews including 44 and 43 cross-sectional studies, respectively [30,31]. One review was assessed to be of high confidence [37], ten of moderate confidence [24,26,29-36] and three of low confidence [25,27,28] (**Table 1** and more detailed appraisal in Table S2 in the **Online Supplementary Document**). Main findings for each systematic review and meta-analysis are summarised in **Table 3**.

**Table 3:** Findings for each diabetes comorbidity from included systematic reviews and meta-analyses

Comorbidity	Author, year	Main findings*	Heterogeneity	Subgroup analysis^†^
Cardiovascular disease	Einarson, 2018 [24]	Weighted average prevalence	N/A	N/A
		*Upper middle-income countries:*		
		Stroke: 6.9%		
		Myocardial infarction: 3.4%		
		Coronary artery disease: 17.7%		
		*Lower middle-income countries:*		
		Stroke: 6.3%		
		Coronary artery disease: 27.3%		
	Poorzand, 2019 [25]	Premature coronary artery disease OR = 2.35 (95% CI = 1.71-3.21)	*I^2^* = 25.5%, *P* = 0.225	N/A
Chronic kidney disease	Koye, 2017 [26]	Annual cumulative incidence in two primary studies: 8.1% and 8.6%	N/A	N/A
	Shiferaw, 2020 [27]	Prevalence, stages 1-5: 36% (95% CI = 26%-45%)	*I^2^*>90%, *P* < 0.001	Study design for stages 3-5
		Prevalence, stages 3-5: 15% (95% CI = 11%-19%)		
Depression	Mendenhall, 2014 [28]	Average prevalence: 36%	N/A	N/A
	Uphoff, 2019 [29]	Prevalence: 40% (95% CI = 34%-45%)	*I^2^* = 97.5%, *P* < 0.001	N/A
	Hussain, 2018 [30]	Prevalence: 38% (95% CI = 31%-45%)	*I^2^*>90%, *P* < 0.001	N/A
	Khalighi, 2019 [31]	Prevalence: 61% (95% CI = 55%-67%)	*I^2^* = 98%, *P* < 0.001	Income level, depression assessment tool
	Wang, 2019 [32]	Prevalence: 30.7% (95% CI = 16.6%-44.9%); *OR = 3.05 (95% CI = 1.11-8.37)*	*I^2^* = 99%, *P* < 0.001; *I^2^* = 90.5%, *P* < 0.001	Study design, income level
Tuberculosis	Bailey & Ayles, 2017 [33]	Adjusted OR range: 2.14 (95% CI = 1.32-3.46) to 19.3 (95% CI = 6.1-61.0)	N/A	N/A
	McMurry, 2018 [34]	Prevalence range: 0.1% to 4.9%	N/A	N/A
	Al-Rifai, 2017 [35]	Risk estimate: 3.40 (95% CI = 1.53-7.56)	*I^2^* = 87.4%, *P* < 0.001	N/A
		OR = 3.04 (95% CI = 2.29-4.03)	*I^2^* = 23.5%, *P* = 0.250	
	Lee, 2017 [36]	OR = 1.16 (95% CI = 0.97-1.37)	*I^2^* = 0%, *P* = 0.608	N/A
	Tegegne, 2018 [37]	OR = 1.78 (95% CI = 1.26-2.52)	*I^2^* = 71.1%, *P* < 0.001	Study design, income level, confounder adjustment

*Prevalence estimates refer to the prevalence of each comorbidity among people with diabetes. Effect estimates refer to the risk or odds of the comorbidity in people with vs without diabetes.

†Subgroup analyses conducted as part of this umbrella review.

### Cardiovascular disease

The two reviews identified for CVD [24,25] included 22 primary studies (six cohort, six case-control, ten cross-sectional) from nine countries. There were 3 108 248 participants, of which 3 060 517 (98%) were from one primary study [39].

Einarson et al [24] did not perform a meta-analysis, but reported that among people with diabetes in upper middle-income countries, the weighted average prevalence of stroke, myocardial infarction, and coronary artery disease was 6.9%, 3.4%, and 17.7%, respectively. In lower middle-income countries the weighted average prevalence of stroke and coronary artery disease was 6.3% and 27.3%, respectively, with no study reporting on the prevalence of myocardial infarction.

**Figure 2:**
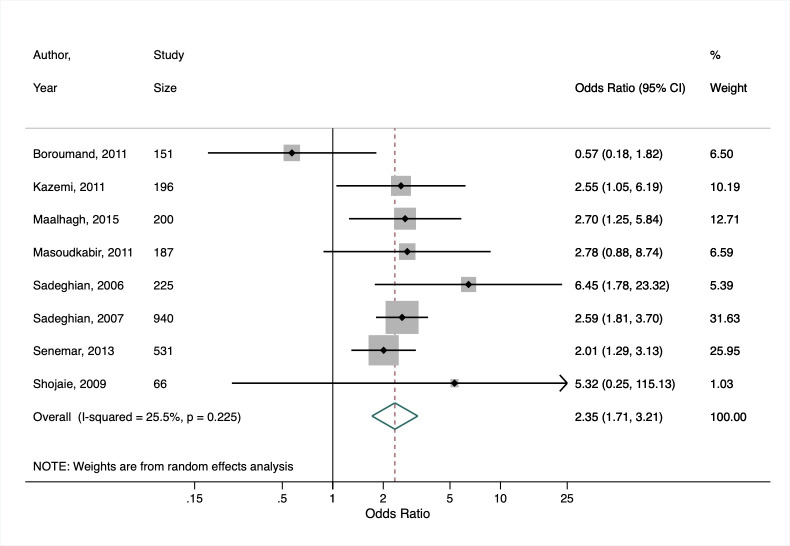
Secondary meta-analysis of studies included in Poorzand et al. [25] showing the study-specific and summary estimates of the prevalence of premature chronic artery disease among people with diabetes in Iran.

Based on our secondary meta-analysis of the eight studies (six case-control, two cross-sectional) from Poorzand et al [25], we found an overall OR for premature coronary artery disease of 2.35 (95% CI = 1.71-3.21) in people with vs without diabetes in Iran, with low heterogeneity between studies (*I^2^* = 25.5%, *P* = 0.225) (**Figure 2**).

### Chronic kidney disease

We identified two reviews [26,27] on CKD occurrence amongst people with diabetes, one of which only included two LMIC studies out of 71 included studies [26], while the other included 12 studies from Ethiopia [27]. The studies from the former were both cohort studies, including a combined total of 1114 participants with diabetes [26]. The annual cumulative incidence of CKD among people with diabetes in each primary study was 8.6% [40] and 8.1% [41].

**Figure 3:**
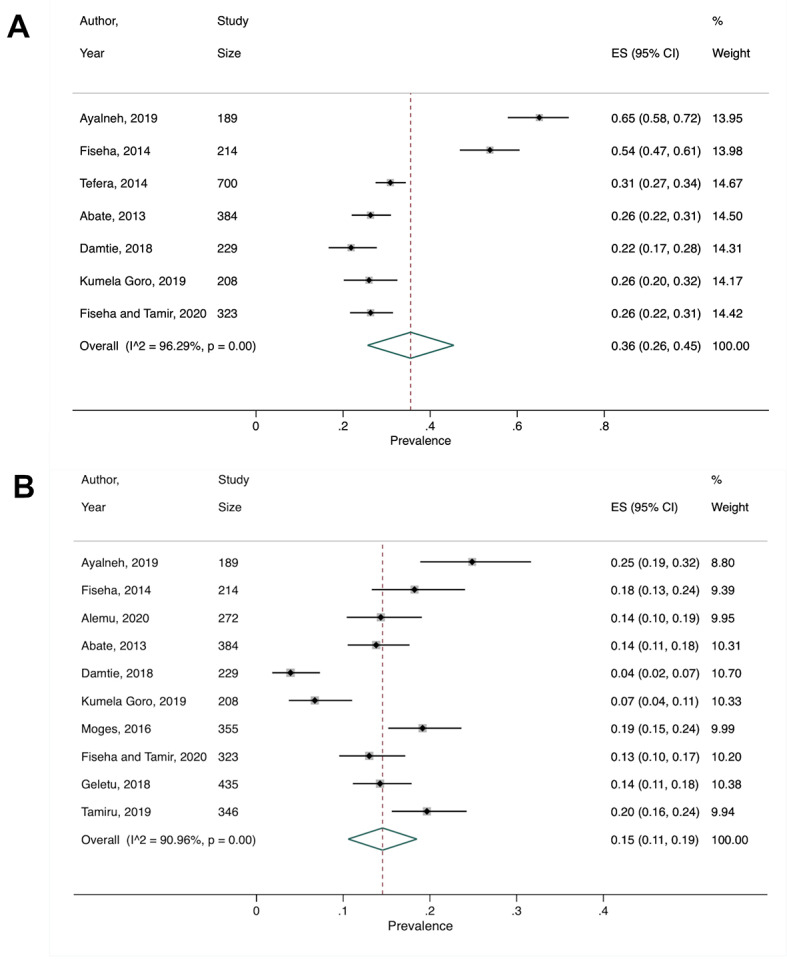
Secondary meta-analysis of studies included in Shiferaw et al. [27] showing the study-specific and summary estimates of the prevalence of chronic kidney disease among people with diabetes in Ethiopia for chronic kidney disease **Panel A.** Stages 1-5. **Panel B.** Stages 3-5.

We performed a secondary meta-analysis on the review by Shiferaw et al, which included two cohort, one case-control, and nine cross-sectional studies [27]. The authors provided prevalence estimates separately for CKD stages 1-5 and 3-5, so we did the same for our secondary meta-analysis. Seven cross-sectional studies were included for stages 1-5 and the pooled prevalence was 36% (95% CI = 26%-45%) (**Figure 3**, Panel A). For stages 3-5, 10 studies gave a pooled CKD prevalence of 15% (95% CI = 11%-19%) (**Figure 3**, Panel B). Heterogeneity for the two groups were both substantial, with I^2^>90%. Income level remained the same for Ethiopia throughout the study period, and all stage 1-5 studies were cross-sectional, which precluded subgroup analyses. Subgroup analysis by study design for stage 3-5 studies was not performed due to having too few studies in each subgroup.

### Depression

Four reviews on depression [28,30-32] included 528 987 participants from 145 primary studies (142 cross-sectional and three case-control). An additional 41 cross-sectional studies, with an unknown number of participants, were from one review which did not provide specific study characteristics [29]. A total of 16 LMICs were represented across all five reviews, with the majority being from India and Iran. Based on the five reviews, we found that in LMICs the average prevalence of depression, including major depressive disorder, in people with diabetes ranged from 30%-61%. We conducted secondary meta-analyses on three of these reviews [30-32].

The systematic review by Mendenhall et al focused specifically on LMICs and found that the average prevalence of depression in people with diabetes was 36% [28]. While Uphoff et al. [29] did conduct a meta-analysis, this was done rapidly so the authors only provided aggregated study characteristics and a pooled depression prevalence. Due to this, we were not able to conduct our own secondary meta-analysis. Nonetheless, based on 43 estimates from 41 cross-sectional studies from Bangladesh, India, and Pakistan, they found a pooled depression prevalence of 40% (95% CI = 34%-45%) in people with diabetes. They found substantial heterogeneity (*I^2^* = 97%) and conducted meta-regression analysis by publication year, country, disease (since they looked at various non-communicable diseases), sample size, diagnostic tool, and study quality. None of these variables fully explained the heterogeneity.

Hussain et al [30] included 43 cross-sectional studies from India and the pooled prevalence from our secondary meta-analysis was 38% (95% CI = 31%-45%). Heterogeneity was substantial (*I^2^* = 99%, *P* < 0.001). Since India remained at the same income level when all the primary studies occurred, we could not do a subgroup analysis using income level.

**Figure 4:**
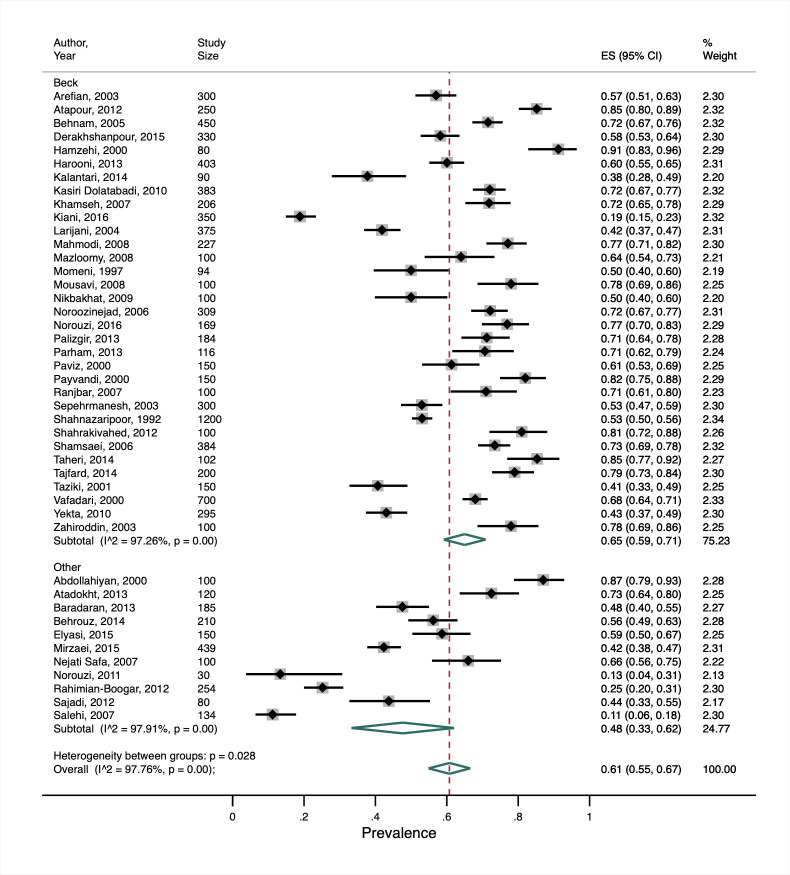
Subgroup analysis from secondary meta-analysis of studies included in Khalighi et al. [31] showing the study-specific and summary estimates of the prevalence of depression in people with diabetes in Iran by depression assessment tool.

Our secondary meta-analysis of Khalighi et al. [31] showed a much higher overall prevalence of depression in people with diabetes (61%, 95% CI = 55%-67%) compared to the other reviews, albeit with substantial heterogeneity (I^2^ = 98%, *P* < 0.001). This review only included studies from Iran, which was considered a lower-middle income country before 2009 and an upper-middle income country from 2009 onwards [17]. Our subgroup analysis did not show any significant difference in the prevalence between the two income-level categorisations. Among the studies identified in this review, eight different depression assessment tools were used, with the Beck Depression Inventory (BDI) the most common, being used in 75% of studies. In a post-hoc subgroup analysis comparing depression prevalence in studies using the BDI vs all other tools, the pooled prevalence was higher in the former than the latter studies (65%, 95% CI = 59%-71% and 48%, 95% CI = 33%-63%, respectively). Whilst between-subgroup heterogeneity was statistically significant (*P* = 0.028), there was still substantial heterogeneity within each subgroup (*I^2^*>97%, *P* < 0.001) (**Figure 4**).

In our secondary meta-analyses of LMIC studies included in the review by Wang et al [32], we computed both the pooled prevalence across all ten studies, and the pooled OR across the three case-control studies. The pooled prevalence of major depressive disorder in people with diabetes was 30.7% (95% CI = 16.6%-44.9%), with substantial heterogeneity between studies (*I^2^* = 99%, *P* < 0.001) (Figure S1 in the **Online Supplementary Document**). Subgroup analyses were not possible given too few studies in each subgroup. The pooled OR for the case-control studies was 3.05 (95% CI = 1.11-8.37), and also had substantial heterogeneity (*I^2^* = 90.5%, *P* < 0.001) (Figure S2 in the **Online Supplementary Document**).

### Tuberculosis

Of the five identified reviews on diabetes and TB [33-37], one focused on latent TB (LTB) [36] and one on multi-drug resistant TB (MDR-TB) [37]. We performed narrative synthesis on two reviews [33,34] and secondary meta-analysis on three [35-37]. In total, these reviews included 300 659 participants from 22 LMICs amongst 47 primary studies (16 cohort, 15 case-control, and 17 cross-sectional). One primary study from McMurry et al [34] reported on two separate cross-sectional studies from different countries, thus both were included in the study count. Generally, diabetes was shown to be associated with an increased risk of developing TB, although the association for LTB was not statistically significant and only included two primary studies.

Bailey and Ayles [33] examined three primary studies on the association between diabetes and TB in African settings, but did not perform a meta-analysis due to between-study heterogeneity. The study specific adjusted ORs from these primary studies suggest that diabetes is associated with increased odds of TB, although effect estimates ranged from 2.14 (95% CI = 1.32-3.46) to 19.3 (95% CI = 6.1-61.0). McMurry et al included 17 primary studies and found that prevalence of TB amongst people with diabetes ranged from 0.1% to 4.9% [34].

**Figure 5:**
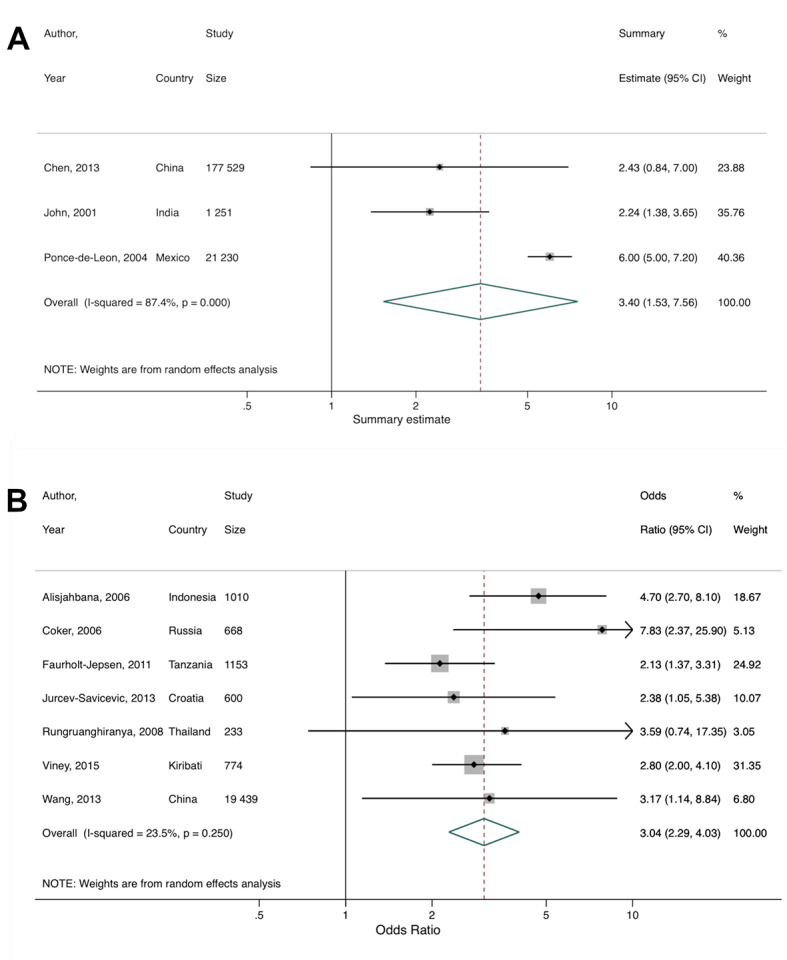
Secondary meta-analysis of studies included in Al-Rifai et al. [35] showing the study-specific and summary estimates of tuberculosis among people with diabetes. **Panel A.** Hazard, risk, and rate. **Panel B.** Odds ratio.

Al-Rifai et al identified nine of their primary studies as being from LMICs, but based on the World Bank income classification used in our review, ten were eligible and thus included in our secondary meta-analysis [35]. Since different effect measures were used across the primary studies, they could not all be combined. After pooling three primary studies which reported a hazard ratio, rate ratio, and relative risk, we found that diabetes was associated with a greater than 3-fold increased risk of TB (summary estimate = 3.40, 95% CI = 1.53-7.56), with substantial heterogeneity between studies (*I^2^* = 87.4%, *P* < 0.001; **Figure 5**, Panel A). Similarly, after pooling seven studies (five case-control, one cross-sectional, and one cohort) reporting odds ratios, we found that diabetes was associated with a 3-fold increased odds of TB (summary OR = 3.04, 95% CI = 2.29-4.03), with low heterogeneity between studies (*I^2^* = 23.5%, *P* = 0.250; **Figure 5**, Panel B). There was no evidence of publication bias (Figure S3 in the **Online Supplementary Document**).

Lee et al [36] only included two cross-sectional studies on the association between diabetes and LTB in LMICs. The pooled summary suggested no significant association between diabetes and LTB (OR = 1.16, 95% CI = 0.97–1.37; heterogeneity: *I^2^* = 0.0%, *P* = 0.608; Figure S4 in the **Online Supplementary Document**).

**Figure 6:**
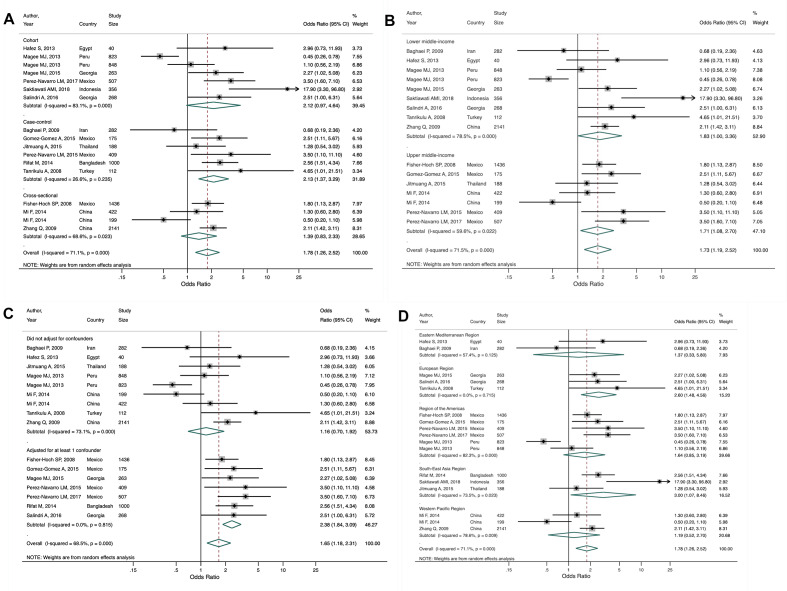
Subgroup analyses from secondary meta-analysis of studies included in Tegegne et al. [37] showing the study-specific and summary estimates of the odds of multi-drug resistant tuberculosis among people with diabetes. **Panel A.** Study design. **Panel B.** Country income level. **Panel C.** Confounder adjustment. **Panel D.** WHO region.

Finally, Tegegne et al. [37] included 17 estimates from 15 studies (six cohort, six case-control, three cross-sectional) from 10 LMICs across all WHO regions. Two studies [42,43] reported estimates separately for those with previously diagnosed TB and newly diagnosed TB. Our secondary meta-analysis of all 17 estimates found that people with vs without diabetes have 78% increased odds (OR = 1.78, 95% CI = 1.26-2.52) of developing MDR-TB, with moderate heterogeneity between studies (*I^2^* = 71.1%, *P* < 0.001). Our study design subgroup analysis found a higher OR and low heterogeneity for case-control studies (OR = 2.13, 95% CI = 1.37-3.29; *I^2^* = 26.6%, *P* = 0.235) compared to cross-sectional and cohort studies (**Figure 6**, Panel A). Our income-level subgroup analysis found similar OR estimates between lower and upper-middle income countries, with substantial and moderate heterogeneity, respectively (**Figure 6**, Panel B). We conducted a post-hoc analysis on confounder adjustment and found that the pooled estimate was higher for those studies that adjusted for at least one confounder, of which age, sex, smoking, and HIV status were the most common, compared to studies that did not adjust for any other factors (OR = 2.57, 95% CI = 1.92-3.43 vs OR = 1.16, 95% CI = 0.70-1.92; **Figure 6**, Panel C). We also conducted a post-hoc analysis by WHO region to see if there may be regional differences. Heterogeneity remained moderate to substantial across regions, with the exception of the European region which showed no heterogeneity (*I^2^* = 0.0%, *P* = 0.715). Only the estimates for South-East Asia and Europe were statistically significant; these regions also had the strongest association between diabetes and MDR-TB (**Figure 6**, Panel D). There was no evidence of publication bias (Figure S5 in the **Online Supplementary Document**).

## DISCUSSION

Our umbrella review identified 14 relevant systematic reviews on the association between diabetes and CVD, CKD, depression, and TB in 29 LMICs, nine of which included meta-analyses. However, no reviews on diabetes and dengue or pneumonia were eligible for inclusion. Whilst there was some evidence for diabetes being associated with each of CVD, CKD, depression, and TB, the extent and quality of evidence varied across comorbidities. The evidence was most robust for the associations between diabetes and CVD and TB, for which there were a number of cohort studies. A lack of studies on the association between diabetes and CKD and the predominance of cross-sectional studies on the association between diabetes and depression reveal gaps in understanding of the association between diabetes and CKD and depression in LMICs, including among many countries for which no data were available.

Compared to findings previously reported from HICs, prevalence and effect estimates for depression and TB were generally higher in LMICs [44-46]. The higher estimates may reflect fewer resources and lower levels of funding for mental health care [47], a higher incidence of TB, and a high percentage of unmet need for diabetes care, in LMICs compared to HICs [48]. Our findings for CVD and CKD were comparable to HICs [7,26,49-52], but this is based on very limited evidence. Further, although this umbrella review specifically searched for reviews from LMICs, only 59.9% of primary studies and 6.42% of the total number of study participants were actually from an LMIC.

Our review has a number of strengths. This is the first umbrella review to systematically synthesise the existing evidence on diabetes and the risk of multiple key comorbidities in LMICs. Our broad scope facilitated the investigation of the potential association between diabetes and both non-communicable and communicable comorbidities, which is particularly relevant to LMIC settings which face a rising dual burden of communicable and non-communicable disease. Our use of secondary meta-analyses provided a new perspective on diabetes comorbidities by focusing specifically on the relationships in LMICs. The review also benefits from having used a detailed, comprehensive electronic search strategy and two reviewers to screen, select and extract data from articles.

This review has several limitations. First, it does not fully represent all the possible comorbidities with which diabetes could be associated. However, the diseases that were included are those commonly associated with diabetes both in LMICs and globally [7,12,53]. Second, given the lack of high-confidence reviews included in this umbrella review, our conclusions are suitably cautious. Third, since primary studies were mainly cross-sectional, conclusions regarding the temporal sequence of diabetes and its comorbidities is limited [54]. However, omitting cross-sectional studies would have disproportionately restricted the scope of our review due to the dearth of longitudinal studies in LMICs. Fourth, secondary meta-analyses could be performed for only nine reviews, and of those only two had sufficient studies to examine publication bias. Finally, meta-analyses found substantial between-study heterogeneity for the associations between diabetes and each of CKD, depression, and TB, the reasons for which are not clear. LMICs are a highly diverse group in terms of their culture, environment, and social determinants of health, and not accounting for these factors may explain the heterogeneity. We were only able to run a subgroup analysis on WHO region for one TB review [37], but it did not explain the observed heterogeneity, and there was insufficient information to explore additional subgroups.

We did not include any reviews on dengue or pneumonia, either due to eligible reviews being of critically low confidence in the case of dengue, or the lack of systematic reviews on the association between diabetes and pneumonia that included primary studies from LMICs. While dengue is more often studied in the context of LMICs compared to the other comorbidities we included, it is not often examined as an outcome of diabetes, which highlights a gap in our understanding of diabetes-dengue comorbidity. The majority of research has viewed diabetes as a risk factor for the progression to more severe forms of dengue, rather than as a risk factor for developing dengue. For example, a recent meta-analysis based on 12 studies (design and country not specified) found that among people who had less severe forms of dengue, diabetes was strongly associated with progression to severe dengue (OR 4.38, 95% CI = 2.58-7.43) [55]. With regard to pneumonia, a previous non-systematic literature review summarised evidence on diabetes and various pneumonias (including TB) globally, but the majority of evidence was for the association between diabetes and TB [56]. The authors acknowledged that there is limited evidence on the relationship between diabetes and pneumococcal, staphylococcal, and influenza pneumonias, and that most of this comes from HICs [56].

Our general conclusion is that more research about diabetes comorbidities needs to be conducted in LMICs, especially for the comorbidities in which we found either no or few primary studies or reviews. Additionally, the bulk of the data identified in our review stems from studies conducted in Iran and India, so findings may not be representative of other settings. This highlights the need for more research in these underrepresented regions to gain a better understanding of the global impact of diabetes and its comorbidities. In particular, there should also be an emphasis on conducting larger, higher-quality, and cohort studies rather than simply increasing the quantity of small cross-sectional studies. However, for this to be done, there needs to be additional funding and resources available for research in LMICs. Once this high-quality primary evidence is available and synthesised in additional systematic reviews and meta-analyses, findings can be used to appropriately inform policy.

Only then will it be possible to base LMIC policies and health priority setting on data that is representative of the LMIC context rather than the HIC context. Most of the disease management guidelines, treatments, and policy recommendations used in LMICs, especially with regard to non-communicable diseases, were either developed in HICs or adapted from existing infectious disease programmes [57]. While LMICs can seek inspiration from strategies that have and have not worked in HICs, it is important that context-specific programmes grounded in relevant data are developed [58]. Additionally, LMICs may be able to learn from HIC experiences of developing and implementing integrated care models. While the structure of health systems, services, and funding varies across LMICs and differs from those in HICs, the horizontal approach that HICs have taken to integrate systems and services, for example for physical and mental health or for people with multiple conditions, may provide valuable examples for LMICs [59]. As this review has identified evidence for CVD, CKD, diabetes, and TB as comorbidities of diabetes, we recommend that these diseases are feasible targets for integrated care and increased screening and monitoring. However, specific recommendations will vary by country due to different disease distributions, health systems, and social structures. In countries with existing HIV programmes, another option could be to integrate additional health services into those programmes, especially as the population living longer with HIV has a higher risk of developing non-communicable diseases [60]. This is particularly relevant as the rising burden of non-communicable diseases, and co- and multi-morbidities, may put further strain on health systems and health care staff that are already overextended and underfunded.

## CONCLUSION

This umbrella review identifies associations between diabetes and a number of key communicable and non-communicable diseases in LMICs. However, a lack of robust evidence, in particular the lack of cohort studies and limited number of participants in these settings, limits conclusions on the extent to which diabetes is associated with particular comorbidities in LMICs and whether associations differ to those in HICs. The shifting of the non-communicable disease burden to LMICs highlights the growing need to fill these gaps in understanding to ensure that policies for primary and secondary prevention can be based on context-specific evidence.

## Additional material

Online Supplementary Document
