# Supplementary material for: Diabetes comorbidities in low- and middle-income countries: An umbrella review
Source: J Glob Health. 2021 Jul 24;11:04040. doi: 10.7189/jogh.11.04040 (PMC8325931; doi:10.7189/jogh.11.04040)
Supplement: Online Supplementary Document [file jogh-11-04040-s001.pdf]

# Appendix S1: Full search strategy for each database

## MEDLINE

Meta-Analysis as Topic/
meta analy$.tw.
metaanaly$.tw.
Meta-Analysis/
(systematic adj (review$1 or overview$1)).tw.
exp Review Literature as Topic/
or/1-6
cochrane.ab.
embase.ab.
(psychlit or psyclit).ab.
(psychinfo or psycinfo).ab.
(cinahl or cinhal).ab.
science citation index.ab.
bids.ab.
cancerlit.ab.
or/8-15
reference list$.ab.
bibliograph$.ab.
hand-search$.ab.
relevant journals.ab.
manual search$.ab.
or/17-21
selection criteria.ab.
data extraction.ab.
23 or 24
Review/
25 and 26
Comment/
Letter/
Editorial/
animal/
human/
31 not (31 and 32)
or/28-30,33
7 or 16 or 22 or 27
35 not 34
Developing Countries.sh,kf.
((developing or less* developed or under developed or underdeveloped or middle income or low* income or underserved or under served or deprived or poor*) adj (countr* or nation? or population? or world)).ti,ab.
((developing or less* developed or under developed or underdeveloped or middle income or low* income) adj (economy or economies)).ti,ab.
(low* adj (gdp or gnp or gross domestic or gross national)).ti,ab.
(low adj3 middle adj3 countr*).ti,ab.
(lmic or lmics or third world or lami countr*).ti,ab.
transitional countr*.ti,ab.
Cambodia/
(cambodia* or Kampuchea).cp,in,jw,mp.
"Democratic People's Republic of Korea"/
(north korea* or (democratic people* republic adj2 korea)).cp,in,jw,mp.
Myanmar/
(myanmar or burma or burmese).cp,in,jw,mp.
Fiji/
fiji*.cp,in,jw,mp.
Indonesia/
indonesia*.cp,in,jw,mp.
Micronesia/
(Micronesia* or Kiribati).cp,in,jw,mp.
Laos/
(laos or (lao adj1 democratic republic) or (lao adj2 people) or marshall island*).cp,in,jw,mp.
Mongolia/
mongolia*.cp,in,jw,mp.
Papua New Guinea/

Papua New Guinea.cp,in,jw,mp.
Philippines/
(Philippines or filipino*).cp,in,jw,mp.
samoa/ or independent state of samoa/
samoa*.cp,in,jw,mp.
Melanesia/
(Solomon Islands or Timor-Leste or Melanesia*).cp,in,jw,mp.
Tonga/
tonga*.cp,in,jw,mp.
Vanuatu/
Vanuatu.cp,in,jw,mp.
Vietnam/
Vietnam*.cp,in,jw,mp.
exp China/
(china or chinese).cp,in,jw,mp.
Malaysia/
Malaysia*.cp,in,jw,mp.
Palau/
(Palau or Belau or Pelew).cp,in,jw,mp.
Thailand/
(Thailand or thai*).cp,in,jw,mp.
(tuvalu or ellice islands).cp,in,jw,mp.
Kyrgyzstan/
(kyrgyzstan or kyrgyz or kirghizia or kirghiz).cp,in,jw,mp.
Tajikistan/
(tajikistan or tadzhik or tadzhikistan or tajikistan).cp,in,jw,mp.
Albania/
Albania*.cp,in,jw,mp.
Armenia/
Armenia*.cp,in,jw,mp.
"Georgia (Republic)"/
georgia*.cp,in,jw,mp.
Yugoslavia/
(Jugoslavija* or Yugoslavia* or serbo-croat* or macedonia* or sloven* or kosovo).cp,in,jw,mp.
Moldova/
Moldova*.cp,in,jw,mp.
Ukraine/
Ukrain*.cp,in,jw,mp.
Uzbekistan/
Uzbekistan.cp,in,jw,mp.
Azerbaijan/
Azerbaijan*.cp,in,jw,mp.
Republic of Belarus/
(belarus or byelarus or belorussia).cp,in,jw,mp.
Bosnia-Herzegovina/
bosnia*.cp,in,jw,mp.
Bulgaria/
Bulgaria*.cp,in,jw,mp.
Kazakhstan/
(Kazakhstan or kazakh).cp,in,jw,mp.
Latvia/
Latvia*.cp,in,jw,mp.
Lithuania/
Lithuania*.cp,in,jw,mp.
"Macedonia (Republic)"/
Macedonia*.cp,in,jw,mp.
Montenegro/
Montenegro.cp,in,jw,mp.
Romania/
Romania*.cp,in,jw,mp.
exp Russia/
USSR/
(russia* or ussr or soviet or cccp).cp,in,jw,mp.
Serbia/
serbia*.cp,in,jw,mp.
Turkey/
turk*.cp,in,jw,mp. not animal/

Turkmenistan/
Haiti/
Haiti.cp,in,jw,mp.
Belize/
Belize.cp,in,jw,mp.
Bolivia/
Bolivia*.cp,in,jw,mp.
El Salvador/
El Salvador.cp,in,jw,mp.
Guatemala/
Guatemala*.cp,in,jw,mp.
Guyana/
Guyana*.cp,in,jw,mp.
Honduras/
Hondura*.cp,in,jw,mp.
Nicaragua/
Nicaragua.cp,in,jw,mp.
Paraguay/
Paraguay.cp,in,jw,mp.
Antigua and Barbuda/
(Antigua or Barbuda).cp,in,jw,mp.
Argentina/
Argentin*.cp,in,jw,mp.
Brazil/
Brazil*.cp,in,jw,mp.
Chile/
Chile*.cp,in,jw,mp.
Colombia/
Colombia*.cp,in,jw,mp.
Costa Rica/
Costa Rica*.cp,in,jw,mp.
Cuba/
Cuba*.cp,in,jw,mp.
Dominica/
Dominican Republic/
Dominica*.cp,in,jw,mp.
Ecuador/
Ecuador*.cp,in,jw,mp.
Grenada/
Grenad*.cp,in,jw,mp.
Jamaica/
Jamaica*.cp,in,jw,mp.
Mexico/
Mexic*.cp,in,jw,mp.
exp Panama/
Panama*.cp,in,jw,mp.
Peru/
Peru*.cp,in,jw,mp.
Saint Lucia/
(St Lucia* or Saint Lucia*).cp,in,jw,mp.
Saint Vincent and the Grenadines/
Grenadines.cp,in,jw,mp.
Suriname/
Surinam*.cp,in,jw,mp.
Uruguay/
Uruguay.cp,in,jw,mp.
Venezuela/
Venezuela*.cp,in,jw,mp.
Djibouti/
Djibouti.cp,in,jw,mp.
Egypt/
Egypt*.cp,in,jw,mp.
Iraq/
Iraq*.cp,in,jw,mp.
Morocco/
Morocc*.cp,in,jw,mp.
Syria/

(Syria* or gaza*).cp,in,jw,mp.
Yemen/
yemen*.cp,in,jw,mp.
Algeria/
Algeria*.cp,in,jw,mp.
Iran/
Iran*.cp,in,jw,mp.
Jordan/
jordan*.cp,in,jw,mp.
Lebanon/
Leban*.cp,in,jw,mp.
Libya/
Libya*.cp,in,jw,mp.
Tunisia/
Tunisia*.cp,in,jw,mp.
Afghanistan/
Afghan*.cp,in,jw,mp.
Bangladesh/
Bangladesh*.cp,in,jw,mp.
Nepal/
Nepal*.cp,in,jw,mp.
Bhutan/
Bhutan*.cp,in,jw,mp.
exp India/
india*.cp,in,jw,mp.
Pakistan/
Pakistan*.cp,in,jw,mp.
Sri Lanka/
Sri Lanka*.cp,in,jw,mp.
Indian Ocean Islands/
Maldiv*.cp,in,jw,mp.
Benin/
(Benin or Dahomey).cp,in,jw,mp.
Burkina Faso/
(Burkina Faso or Burkina Fasso or Upper Volta).cp,in,jw,mp.
Burundi/
Burundi*.cp,in,jw,mp.
Central African Republic/
(Central African Republic or Ubangi-Shari or african*).cp,in,jw,mp.
Chad/
Chad.cp,in,jw,mp.
Comoros/
(comoros or comores).cp,in,jw,mp.
Democratic Republic of the Congo/
(congo* or zaire).cp,in,jw,mp.
Eritrea/
Eritrea*.cp,in,jw,mp.
Ethiopia/
Ethiopia*.cp,in,jw,mp.
Gambia/
Gambia*.cp,in,jw,mp.
Guinea/
(Guinea* not (New Guinea or Guinea Pig* or Guinea Fowl)).cp,in,jw,mp.
Guinea-Bissau/
(Guinea-Bissau or Portuguese Guinea).cp,in,jw,mp.
Kenya/
Kenya*.cp,in,jw,mp.
Liberia/
Liberia*.cp,in,jw,mp.
Madagascar/
(Madagasca* or Malagasy Republic).cp,in,jw,mp.
Malawi/
(Malawi* or Nyasaland).cp,in,jw,mp.
Mali/
Mali*.cp,in,jw,mp.
Mauritania/
Mauritania*.cp,in,jw,mp.

Mozambique/
(Mozambi* or Portuguese East Africa).cp,in,jw,mp.
Niger/
(Niger not (Aspergillus or Peptococcus or Schizothorax or Cruciferae or Gobius or Lasius or Agelastes or Melanosuchus or radish or Parastromateus or Orius or Apergillus or Parastromateus or Stomoxys)).cp,in,jw,mp.
Rwanda/
(Rwanda* or Ruanda*).cp,in,jw,mp.
Sierra Leone/
Sierra Leone*.cp,in,jw,mp.
Somalia/
Somali*.cp,in,jw,mp.
Tanzania/
Tanzania*.cp,in,jw,mp.
Togo/
Togo*.cp,in,jw,mp.
Uganda/
Uganda*.cp,in,jw,mp.
Zimbabwe/
(Zimbabwe* or Rhodesia*).cp,in,jw,mp.
Cameroon/
Cameroon*.cp,in,jw,mp.
Cape Verde/
Cape Verde*.cp,in,jw,mp.
Congo/
(congo* not ((democratic republic adj congo) or congo red or crimean-congo)).cp,in,jw,mp.
Cote dIvoire/
(Cote dIvoire or Ivory Coast).cp,in,jw,mp.
Ghana/
(Ghan* or Gold Coast).cp,in,jw,mp.
Lesotho/
(Lesotho or Basutoland).cp,in,jw,mp.
Nigeria/
Nigeria*.cp,in,jw,mp.
Atlantic Islands/
(sao tome adj principe).cp,in,jw,mp.
Senegal/
Senegal*.cp,in,jw,mp.
Sudan/
Sudan*.cp,in,jw,mp.
Swaziland/
Swazi*.cp,in,jw,mp.
Zambia/
(Zambia* or Northern Rhodesia*).cp,in,jw,mp.
Angola/
Angola*.cp,in,jw,mp.
Botswana/
(Botswana* or Bechuanaland or Kalahari).cp,in,jw,mp.
Gabon/
Gabon*.cp,in,jw,mp.
Mauritius/
(Mauriti* or Agalega Islands).cp,in,jw,mp.
Namibia/
Namibia*.cp,in,jw,mp.
Seychelles/
Seychelles.cp,in,jw,mp.
South Africa/
South Africa*.cp,in,jw,mp.
or/37-317
exp diabetes mellitus/ or exp hyperglycemia/
dysglyc?mia.mp.
diabetes.mp.
319 or 320 or 321
exp tuberculosis/
(tuberculosis or TB).mp.
exp dengue/
(dengue fever or dengue h?morrhagic fever or dengue shock syndrome or severe dengue).mp.
exp pneumonia/

pneumonia.mp.
exp depression/
exp depressive disorder/
exp renal insufficiency/
(chronic kidney disease or chronic kidney failure or chronic renal insufficiency or kidney dysfunction).mp.
exp cardiovascular diseases/
exp myocardial ischemia/
isch?mic heart disease.mp.
myocardial infarction.mp.
angina.mp.
(coronary heart disease or coronary disease or coronary artery disease).mp.
cerebrovascular disorders/
stroke/
stroke.mp.
Ischemic Attack, Transient/
or/ 323-342
322 and 343
36 and 318 and 344

## EMBASE

exp Meta Analysis/
((meta adj analy$) or metaanalys$).tw.
(systematic adj (review$1 or overview$1)).tw.
or/1-3
cancerlit.ab.
cochrane.ab.
embase.ab.
(psychlit or psyclit).ab.
(psychinfo or psycinfo).ab.
(cinahl or cinhal).ab.
science citation index.ab.
bids.ab.
or/5-12
reference lists.ab.
bibliograph$.ab.
hand-search$.ab.
manual search$.ab.
relevant journals.ab.
or/14-18
data extraction.ab.
selection criteria.ab.
20 or 21
review.pt.
22 and 23
letter.pt.
editorial.pt.
animal/
human/
27 not (27 and 28)
or/25-26,29
4 or 13 or 19 or 24
31 not 30
developing country.sh,hw.
((developing or less* developed or under developed or underdeveloped or middle income or low* income or underserved or under served or deprived or poor*) adj (countr* or nation? or population? or world)).ti,ab.
((developing or less* developed or under developed or underdeveloped or middle income or low* income) adj (economy or economies)).ti,ab.
(low* adj (gdp or gnp or gross domestic or gross national)).ti,ab.
(low adj3 middle adj3 countr*).ti,ab.
(lmic or lmics or third world or lami countr*).ti,ab.
transitional countr*.ti,ab.
Cambodia/
(cambodia* or Kampuchea).cp,in,jw,mp.
"Democratic People's Republic of Korea"/
(north korea* or (democratic people* republic adj2 korea)).cp,in,jw,mp.

Myanmar/
(myanmar or burma or burmese).cp,in,jw,mp.
Fiji/
fiji*.cp,in,jw,mp.
Indonesia/
indonesia*.cp,in,jw,mp.
Micronesia/
(Micronesia* or Kiribati).cp,in,jw,mp.
Laos/
(laos or (lao adj1 democratic republic) or (lao adj2 people) or marshall island*).cp,in,jw,mp.
Mongolia/
mongolia*.cp,in,jw,mp.
Papua New Guinea/
Papua New Guinea.cp,in,jw,mp.
Philippines/
(Philippines or filipino*).cp,in,jw,mp.
samoa/ or "independent state of samoa"/
samoa*.cp,in,jw,mp.
Melanesia/
(Solomon Islands or Timor-Leste or Melanesia*).cp,in,jw,mp.
Tonga/
tonga*.cp,in,jw,mp.
Vanuatu/
Vanuatu.cp,in,jw,mp.
Vietnam/
Vietnam*.cp,in,jw,mp.
exp China/
(china or chinese).cp,in,jw,mp.
Malaysia/
Malaysia*.cp,in,jw,mp.
Palau/
(Palau or Belau or Pelew).cp,in,jw,mp.
Thailand/
(Thailand or thai*).cp,in,jw,mp.
(tuvalu or ellice islands).cp,in,jw,mp.
Kyrgyzstan/
(kyrgyzstan or kyrgyz or kirghizia or kirghiz).cp,in,jw,mp.
Tajikistan/
(tajikistan or tadzhik or tadzhikistan or tajikistan).cp,in,jw,mp.
Albania/
Albania*.cp,in,jw,mp.
Armenia/
Armenia*.cp,in,jw,mp.
"Georgia (Republic)"/
georgia*.cp,in,jw,mp.
Yugoslavia/
(Jugoslavija* or Yugoslavia* or serbo-croat* or macedonia* or sloven* or kosovo).cp,in,jw,mp.
Moldova/
Moldova*.cp,in,jw,mp.
Ukraine/
Ukrain*.cp,in,jw,mp.
Uzbekistan/
Uzbekistan.cp,in,jw,mp.
Azerbaijan/
Azerbaijan*.cp,in,jw,mp.
"Republic of Belarus"/
(belarus or byelarus or belorussia).cp,in,jw,mp.
Bosnia-Herzegovina/
bosnia*.cp,in,jw,mp.
Bulgaria/
Bulgaria*.cp,in,jw,mp.
Kazakhstan/
(Kazakhstan or kazakh).cp,in,jw,mp.
Latvia/
Latvia*.cp,in,jw,mp.
Lithuania/
Lithuania*.cp,in,jw,mp.

"Macedonia (Republic)"/
Macedonia*.cp,in,jw,mp.
Montenegro/
Montenegro.cp,in,jw,mp.
Romania/
Romania*.cp,in,jw,mp.
exp Russia/
USSR/
(russia* or ussr or soviet or cccp).cp,in,jw,mp.
Serbia/
serbia*.cp,in,jw,mp.
Turkey/
turk*.cp,in,jw,mp. not animal/
Turkmenistan/
Haiti/
Haiti.cp,in,jw,mp.
Belize/
Belize.cp,in,jw,mp.
Bolivia/
Bolivia*.cp,in,jw,mp.
El Salvador/
El Salvador.cp,in,jw,mp.
Guatemala/
Guatemala*.cp,in,jw,mp.
Guyana/
Guyana*.cp,in,jw,mp.
Honduras/
Hondura*.cp,in,jw,mp.
Nicaragua/
Nicaragua.cp,in,jw,mp.
Paraguay/
Paraguay.cp,in,jw,mp.
"Antigua and Barbuda"/
(Antigua or Barbuda).cp,in,jw,mp.
Argentina/
Argentin*.cp,in,jw,mp.
Brazil/
Brazil*.cp,in,jw,mp.
Chile/
Chile*.cp,in,jw,mp.
Colombia/
Colombia*.cp,in,jw,mp.
Costa Rica/
Costa Rica*.cp,in,jw,mp.
Cuba/
Cuba*.cp,in,jw,mp.
Dominica/
Dominican Republic/
Dominica*.cp,in,jw,mp.
Ecuador/
Ecuador*.cp,in,jw,mp.
Grenada/
Grenad*.cp,in,jw,mp.
Jamaica/
Jamaica*.cp,in,jw,mp.
Mexico/
Mexic*.cp,in,jw,mp.
exp Panama/
Panama*.cp,in,jw,mp.
Peru/
Peru*.cp,in,jw,mp.
Saint Lucia/
(St Lucia* or Saint Lucia*).cp,in,jw,mp.
"Saint Vincent and the Grenadines"/
Grenadines.cp,in,jw,mp.
Suriname/
Surinam*.cp,in,jw,mp.

Uruguay/
Uruguay.cp,in,jw,mp.
Venezuela/
Venezuela*.cp,in,jw,mp.
Djibouti/
Djibouti.cp,in,jw,mp.
Egypt/
Egypt*.cp,in,jw,mp.
Iraq/
Iraq*.cp,in,jw,mp.
Morocco/
Morocc*.cp,in,jw,mp.
Syria/
(Syria* or gaza*).cp,in,jw,mp.
Yemen/
yemen*.cp,in,jw,mp.
Algeria/
Algeria*.cp,in,jw,mp.
Iran/
Iran*.cp,in,jw,mp.
Jordan/
jordan*.cp,in,jw,mp.
Lebanon/
Leban*.cp,in,jw,mp.
Libya/
Libya*.cp,in,jw,mp.
Tunisia/
Tunisia*.cp,in,jw,mp.
Afghanistan/
Afghan*.cp,in,jw,mp.
Bangladesh/
Bangladesh*.cp,in,jw,mp.
Nepal/
Nepal*.cp,in,jw,mp.
Bhutan/
Bhutan*.cp,in,jw,mp.
exp India/
india*.cp,in,jw,mp.
Pakistan/
Pakistan*.cp,in,jw,mp.
Sri Lanka/
Sri Lanka*.cp,in,jw,mp.
Indian Ocean Islands/
Maldiv*.cp,in,jw,mp.
Benin/
(Benin or Dahomey).cp,in,jw,mp.
Burkina Faso/
(Burkina Faso or Burkina Fasso or Upper Volta).cp,in,jw,mp.
Burundi/
Burundi*.cp,in,jw,mp.
Central African Republic/
(Central African Republic or Ubangi-Shari or african*).cp,in,jw,mp.
Chad/
Chad.cp,in,jw,mp.
Comoros/
(comoros or comores).cp,in,jw,mp.
"Democratic Republic of the Congo"/
(congo* or zaire).cp,in,jw,mp.
Eritrea/
Eritrea*.cp,in,jw,mp.
Ethiopia/
Ethiopia*.cp,in,jw,mp.
Gambia/
Gambia*.cp,in,jw,mp.
Guinea/
(Guinea* not (New Guinea or Guinea Pig* or Guinea Fowl)).cp,in,jw,mp.
Guinea-Bissau/

(Guinea-Bissau or Portuguese Guinea).cp,in,jw,mp.
Kenya/
Kenya*.cp,in,jw,mp.
Liberia/
Liberia*.cp,in,jw,mp.
Madagascar/
(Madagasca* or Malagasy Republic).cp,in,jw,mp.
Malawi/
(Malawi* or Nyasaland).cp,in,jw,mp.
Mali/
Mali*.cp,in,jw,mp.
Mauritania/
Mauritania*.cp,in,jw,mp.
Mozambique/
(Mozambi* or Portuguese East Africa).cp,in,jw,mp.
Niger/
(Niger not (Aspergillus or Peptococcus or Schizothorax or Cruciferae or Gobius or Lasius or Agelastes or Melanosuchus or radish or Parastromateus or Orius or Apergillus or Parastromateus or Stomoxys)).cp,in,jw,mp.
Rwanda/
(Rwanda* or Ruanda*).cp,in,jw,mp.
Sierra Leone/
Sierra Leone*.cp,in,jw,mp.
Somalia/
Somali*.cp,in,jw,mp.
Tanzania/
Tanzania*.cp,in,jw,mp.
Togo/
Togo*.cp,in,jw,mp.
Uganda/
Uganda*.cp,in,jw,mp.
Zimbabwe/
(Zimbabwe* or Rhodesia*).cp,in,jw,mp.
Cameroon/
Cameroon*.cp,in,jw,mp.
Cape Verde/
Cape Verde*.cp,in,jw,mp.
Congo/
(congo* not ((democratic republic adj3 congo) or congo red or crimean-congo)).cp,in,jw,mp.
Cote d'Ivoire/
(Cote d'Ivoire or Ivory Coast).cp,in,jw,mp.
Ghana/
(Ghan* or Gold Coast).cp,in,jw,mp.
Lesotho/
(Lesotho or Basutoland).cp,in,jw,mp.
Nigeria/
Nigeria*.cp,in,jw,mp.
Atlantic Islands/
(sao tome adj2 principe).cp,in,jw,mp.
Senegal/
Senegal*.cp,in,jw,mp.
Sudan/
Sudan*.cp,in,jw,mp.
Swaziland/
Swazi*.cp,in,jw,mp.
Zambia/
(Zambia* or Northern Rhodesia*).cp,in,jw,mp.
Angola/
Angola*.cp,in,jw,mp.
Botswana/
(Botswana* or Bechuanaland or Kalahari).cp,in,jw,mp.
Gabon/
Gabon*.cp,in,jw,mp.
Mauritius/
(Mauriti* or Agalega Islands).cp,in,jw,mp.
Namibia/
Namibia*.cp,in,jw,mp.
Seychelles/

Seychelles.cp,in,jw,mp.
South Africa/
South Africa*.cp,in,jw,mp.
or/33-313
exp diabetes mellitus/ or exp hyperglycemia/
exp dysglycemia/
diabetes.mp.
315 or 316 or 317
exp tuberculosis/
(tuberculosis or TB).mp.
exp dengue/
(dengue fever or dengue h?morrhagic fever or dengue shock syndrome or severe dengue).mp.
exp pneumonia/
pneumonia.mp.
exp depression/
depressive disorder.mp.
exp chronic kidney failure/
(chronic kidney disease or chronic kidney failure or chronic renal insufficiency or kidney dysfunction).mp.
exp cardiovascular disease/
exp ischemic heart disease/
isch?mic heart disease.mp.
coronary heart disease.mp.
coronary artery disease.mp.
heart infarction/
myocardial infarction.mp.
angina pectoris/
angina.mp.
cerebrovascular disease/
cerebrovascular accident/
transient isch?mic attack/
stroke.mp.
or/ 319-341
318 and 342
32 and 314 and 343

## GLOBAL HEALTH

exp meta-analysis/ or exp systematic reviews/
((meta adj analy$) or metaanalys$).tw.
(systematic adj (review$1 or overview$1)).tw.
or/1-3
reference lists.ab.
bibliograph$.ab.
hand-search$.ab.
manual search$.ab.
relevant journals.ab.
or/5-9
data extraction.ab.
selection criteria.ab.
11 or 12
review.tw,ab.
13 and 14
4 or 10 or 15
exp diabetes/ or exp diabetes mellitus/
exp hyperglycaemia/
(diabetes or diabetes mellitus).mp.
17 or 18 or 19
exp tuberculosis/
(TB or tuberculosis).mp.
exp dengue haemorrhagic fever/ or exp dengue shock syndrome/ or exp dengue virus/
(dengue fever or dengue h?morrhagic fever or dengue shock syndrome or severe dengue).mp.
exp pneumonia/
pneumonia.mp.
exp depression/
(depression or depressive disorder).mp.
exp kidney diseases/

(chronic kidney disease or chronic kidney failure or chronic renal insufficiency or kidney dysfunction).mp.
exp cardiovascular diseases/
myocardial ischaemia/
myocardial infarction/
isch?mic heart disease.mp.
angina.mp.
coronary heart disease.mp.
coronary artery disease.mp.
cerebrovascular disorders/
stroke/
stroke.mp.
transient isch?mic attack.mp.
or/21-41
20 and 42
16 and 43

## GLOBAL INDEX MEDICUS

tw:((tw:(tuberculosis OR tb OR dengue OR pneumonia OR depression OR depressive disorder OR kidney disease OR renal insufficiency OR kidney dysfunction OR cardiovascular disease OR cvd OR cerebrovascular disorder OR stroke OR myocardial ischemia OR coronary disease OR ischemic attack OR tia OR mi OR myocardial infarction OR angina)) AND (tw:(diabetes OR hyperglycemia OR hyperglycaemia)) AND (tw:(meta-analysis OR meta analysis OR systematic review)))

Filtered by databases: "LILACS" OR "WPRIM" OR "IMEMR" OR "IMSEAR"

## Appendix S2: Adapted AMSTAR 2 quality assessment tool

### AMSTAR 2 Critical Appraisal Tool

*Adapted for use for systematic reviews on observational studies*

**Title:** ______________________________________________

**Author(s):** ______________________________________________

| | | |
|---|---|---|
| **1. Did the research questions and inclusion criteria for the review include the components of PICO?** | | |
| ☐ Yes<br>☐ No | If yes:<br>☐ Population: ________________<br>☐ Exposure: ________________<br>☐ Comparator group: ____________<br>☐ Outcome: ________________ | Optional (recommended):<br>☐ Timeframe for follow-up |
| **2. Did the report of the review contain an explicit statement that the review methods were established prior to the conduct of the review and did the report justify any significant deviations from the protocol?** | | |
| ☐ Yes<br>☐ Partial Yes<br>☐ No | If partial yes:<br>The authors state that they had a written protocol or guide that included ALL the following:<br>☐ Review question(s)<br>☐ A search strategy<br>☐ Inclusion/exclusion criteria<br>☐ A risk of bias assessment | If yes:<br>As for partial yes, plus the protocol should be registered and should also have specified:<br>☐ A meta-analysis/synthesis plan, if appropriate, *and*<br>☐ A plan for investigating causes of heterogeneity<br>☐ Justification for any deviations from the protocol |
| **3. Did the review authors explain their selection of the study designs for inclusion in the review?** | | |
| ☐ Yes<br>☐ No | If yes, the review should satisfy ONE of the following:<br>☐ *Explanation for* including only one type of observational study (i.e. cross-sectional, case-control, or cohort)<br>☐ OR *explanation for* including a mixture of observational studies | |
| **4. Did the review authors use a comprehensive literature search strategy?** | | |
| ☐ Yes<br>☐ Partial Yes<br>☐ No | If partial yes (all the following):<br>☐ Searched at least 2 databases (relevant to research question)<br>☐ Provided key word and/or search strategy<br>☐ Justified publication restrictions (e.g. language) | If yes, should also have (all the following):<br>☐ Searched the reference lists/ bibliographies of included studies<br>☐ Searched trial/study registries<br>☐ Included/consulted content experts in the field<br>☐ Where relevant, searched for grey literature<br>☐ Conducted search within 24 months of completion of review |
| **5. Did the review authors perform study selection in duplicate?** | | |
| ☐ Yes<br>☐ No | If yes, either ONE of the following:<br>☐ At least two reviewers independently agreed on selection of eligible studies and achieved consensus on which studies to include<br>☐ OR two reviewers selected a sample of eligible studies <u>and</u> achieved good agreement (at least 80%), with the remainder selected by one reviewer | |
| **6. Did the review authors perform data extraction in duplicate?** | | |

| | | |
|---|---|---|
| ☐ Yes<br>☐ No | If yes, either ONE of the following:<br>☐ At least two reviewers achieved consensus on which data to extract from included studies<br>☐ OR two reviewers extracted data from a sample of eligible studies <u>and</u> achieved good agreement (at least 80%), with the remainder extracted by one reviewer | |
| **7. Did the review authors provide a list of excluded studies and justify the exclusions?** | | |
| ☐ Yes<br>☐ Partial Yes<br>☐ No | If partial yes:<br>☐ Provided a list of all potentially relevant studies that were read in full-text form but excluded from the review | If yes, must also have:<br>☐ Justified the exclusion from the review of each potentially relevant study |
| **8. Did the review authors describe the included studies in adequate detail?** | | |
| ☐ Yes<br>☐ Partial Yes<br>☐ No | If partial yes (all the following):<br>☐ Described populations<br>☐ Described exposure<br>☐ Described comparators<br>☐ Described outcomes<br>☐ Described research designs | If yes, should also have all the following:<br>☐ Described population in detail<br>☐ Described exposure in detail<br>☐ Described comparator in detail<br>☐ Described study setting(s)<br>☐ Timeframe for follow-up |
| **9. Did the review authors use a satisfactory technique for assessing the risk of bias (RoB) in individual studies that were included in the review?** | | |
| ☐ Yes<br>☐ Partial Yes<br>☐ No | If partial yes, must have assessed RoB:<br>☐ From confounding (i.e. were appropriate methods done to address possible confounding), *and*<br>☐ From selection bias | If yes, must also have assessed RoB:<br>☐ Methods used to ascertain exposures and outcomes (i.e. measurement bias), *and*<br>☐ Pre-specified selection of the reported result from among multiple measurements or analyses of an outcome (should be specified in protocol) |
| **10. Did the review authors describe any quality appraisal performed on the individual studies that were included in the review?** | | |
| ☐ Yes<br>☐ Partial Yes<br>☐ No | If partial yes (all the following):<br>☐ Described that quality appraisal was performed for each study<br>☐ Described what quality appraisal tool was used *and* provide justification for why that tool was chosen | If yes, should also have all the following:<br>☐ Quality descriptions for each individual study included |
| **11. Did the review authors report on the sources of funding for the studies included in the review?** | | |
| ☐ Yes<br>☐ No | If yes:<br>☐ Must have reported on the sources of funding for individual studies included in the review. *Note: reporting that the reviewers looked for this information, but it was not reported by study authors also qualifies* | |
| **12. If meta-analysis was performed, did the review authors use appropriate methods for statistical combination of results?** | | |
| ☐ Yes<br>☐ No<br>☐ No meta-analysis conducted | If yes:<br>☐ The authors justified combining the data in a meta-analysis<br>☐ AND they used an appropriate weighted technique (i.e. fixed or random effects models) to combine study results, adjusting for heterogeneity, if present<br>☐ AND they statistically combined effect estimates that were adjusted for confounding, rather than combining raw data, or justified combining raw data when adjusted effect estimates were not available | |

| | |
|---|---|
| **13. If meta-analysis was performed, did the review authors assess the potential impact of RoB in individual studies on the results of the meta-analysis or other evidence synthesis?** | |
| ☐ Yes<br>☐ No<br>☐ No meta-analysis conducted | If yes:<br>☐ If the pooled estimate was based on observational studies at variable RoB, the authors performed analyses to investigate possible impact of RoB on summary estimates of effect |
| **14. Did the review authors account for RoB in individual studies when interpreting/discussing the results of the review?** | |
| ☐ Yes<br>☐ No | If yes:<br>☐ Provided a discussion of the likely impact of RoB on the results |
| **15. Did the review authors provide a satisfactory explanation for, and discussion of, any heterogeneity observed in the results of the review?** | |
| ☐ Yes<br>☐ No | If yes:<br>☐ There was no significant heterogeneity in the results<br>☐ OR if heterogeneity was present, the authors performed an investigation of sources of any heterogeneity in the results and discussed the impact of this on the results |
| **16. If they performed quantitative synthesis, did the review authors carry out an adequate investigation of publication bias (small study bias) and discuss its likely impact on the results?** | |
| ☐ Yes<br>☐ No<br>☐ No meta-analysis conducted | If yes:<br>☐ Performed graphical or statistical tests for publication bias and discussed the likelihood and magnitude of impact of publication bias |
| **17. Did the review authors report any potential sources of conflict of interest, including any funding they received for conducting the review?** | |
| ☐ Yes<br>☐ No | If yes:<br>☐ The authors reported no competing interests OR<br>☐ The authors described their funding sources and how they managed potential conflicts of interest |

## Table S1: Articles excluded after full-text screening and quality assessment

## Excluded after full-text screening (n=110)

| Reference | Reason for Exclusion |
|---|---|
| Aambø A and Klemsdal TO. Cardiovascular disease and diabetes in patients with African or Asian background. Tidsskr Nor Legeforen 2017; 137: 28. Review. DOI: https://dx.doi.org/10.4045/tidsskr.16.0680. | HICs |
| Abd ElHafeez S, D'Arrigo G, Bolignano D, et al. The burden of CKD in high risk conditions in the African continent: A systematic review. Nephrology Dialysis Transplantation 2015; 30(Supplement 3): iii194. Conference Abstract. DOI: 10.1093/ndt/gfv175.58. | Abstract |
| Abd ElHafeez S, D'Arrigo G, Bolignano D, et al. The CKD Epidemic in the African Continent: A systematic review. Nephrology Dialysis Transplantation 2015; 30(Supplement 3)): iii505-iii506. Conference Abstract. DOI: 10.1093/ndt/gfv192.49. | Abstract |
| Abd ElHafeez S, Bolignano D, D'Arrigo G, Dounousi E, Tripepi G and Zoccali C. Prevalence and burden of chronic kidney disease among the general population and high-risk groups in Africa: a systematic review. BMJ Open 2018; 8: e015069. DOI: 10.1136/bmjopen-2016-015069. | Did not meet inclusion criteria |
| Abu-Ashour W, Twells L, Valcour J, Randell A, Donnan J, Howse P, et al. The association between diabetes mellitus and incident infections: a systematic review and meta-analysis of observational studies. *BMJ Open Diabetes Res Care* 2017; 5: e000336. DOI: 10.1136/bmjdrc-2016-000336. | Did not meet inclusion criteria |
| Abu Zaid H and Mahajan PS. Influence of Diabetes Mellitus on Stroke. British Journal of Medicine and Medical Research 2016; 12: 1-10. DOI: 10.9734/bjmmr/2016/22937. | Not systematic review |
| Acs A, Ludwig C, Bereza BG, et al. Prevalence of cardiovascular disease in type 2 diabetes: A global systematic review. Value in Health 2017; 20 (9): A475. Conference Abstract. DOI: 10.1016/j.jval.2017.08.435. | Abstract |
| Ahlawat R, Tiwari P, D'Cruz S, et al. Prevalence of chronic kidney disease in India: A systematic review and meta-analysis of observational studies. Value in Health 2015; 18 (7): A509. Conference Abstract. DOI: https://doi.org/10.1016/j.jval.2015.09.1461. | Abstract |
| Ali S, Stone MA, Peters JL, et al. The prevalence of co-morbid depression in adults with Type 2 diabetes: a systematic review and meta-analysis. Diabetic Medicine 2006; 23: 1165-1173. DOI: 10.1111/j.1464-5491.2006.01943.x. | HICs |
| Alizadeh S, Ahmadi M, Ghorbani Nejad B, Djazayeri A and Shab-Bidar S. Metabolic syndrome and its components are associated with increased chronic kidney disease risk: Evidence from a meta-analysis on 11 109 003 participants from 66 studies. *Int J Clin Pract* 2018: e13201. DOI: 10.1111/ijcp.13201. | Did not meet inclusion criteria |
| Almirall J, Serra-Prat M, Bolibar I and Balasso V. Risk Factors for Community-Acquired Pneumonia in Adults: A Systematic Review of Observational Studies. *Respiration* 2017; 94: 299-311. DOI: 10.1159/000479089. | HICs |
| Alzoubi A, Abunaser R, Khassawneh A, et al. The Bidirectional Relationship between Diabetes and Depression: A Literature Review. Korean J Fam Med 2018; 39: 137-146. 2018/05/24. DOI: 10.4082/kjfm.2018.39.3.137. | Not systematic review |
| Alzueta GC and Ezcurra MC. Neumonía adquirida en la comunidad y diabetes: Revisión del tema y propuesta de pautas para tratamiento antibiótico empírico inicial e inmunización. *Revista de la Sociedad Argentina de Diabetes* 2000; 34: 13-20. | Not systematic review |
| Anderson RJ, Freedland KE, Clouse RE, et al. The prevalence of comorbid depression in adults with diabetes: a meta-analysis. Diabetes Care 2001; 24: 1069-1078. 2001/05/26. DOI: 10.2337/diacare.24.6.1069. | HICs |
| Anothaisintawee T, Rattanasiri S, Ingsathit A, et al. Prevalence of chronic kidney disease: a systematic review and meta-analysis. Clinical Nephrology 2009; 71: 244-254. DOI: 10.5414/CNP71244. | HICs |
| Arnold Y, Licea M and Castelo L. Diabetes mellitus y tuberculosis. *Revista Peruana de Epidemiología* 2012; 16: 76-83. | Not systematic review |
| Aston SJ and Rylance J. Community-Acquired Pneumonia in Sub-Saharan Africa. *Semin Respir Crit Care Med* 2016; 37: 855-867. DOI: 10.1055/s-0036-1592126. | Did not meet inclusion criteria |
| Azami M, Moslemirad M, Mansouri A, Khataee M and Sayehmiri K. The Prevalence of Depression in Patients with Diabetes in Iran. *J Babol Univ Med Sci* 2017; 19: 16-27. | Overlapping primary studies |
| Badawi A, Velummailum R, Ryoo SG, et al. Prevalence of chronic comorbidities in dengue fever and West Nile virus: A systematic review and meta-analysis. PLoS One 2018; 13: e0200200. 2018/07/11. DOI: 10.1371/journal.pone.0200200. | Diabetes as outcome |
| Bădescu S, Tătaru C, Kobylinska L, Georgescu E, Zahiu D, Zăgrean A, et al. The association between Diabetes mellitus and Depression. *Journal of Medicine and Life* 2016; 9: 120-125. | Not systematic review |

| | |
|---|---|
| Bekele BB. The prevalence of macro and microvascular complications of DM among patients in Ethiopia 1990-2017: Systematic review. Diabetes Metab Syndr 2019; 13: 672-677. 2019/01/16. DOI: 10.1016/j.dsx.2018.11.046. | Did not meet inclusion criteria |
| Benamer HT and Grosset D. Stroke in Arab countries: A systematic literature review. Journal of the Neurological Sciences 2009; 284: 18-23. Review. DOI: 10.1016/j.jns.2009.04.029. | Did not meet inclusion criteria |
| Berenzon S, Lara MA, Robles R and Medina-Mora ME. Depresión: estado del conocimiento y la necesidad de políticas públicas y planes de acción en México. *Salud Pública México* 2013; 55: 74-80. | Not systematic review |
| Betônico CC, Titan SM, Correa-Giannella ML, Nery M and Queiroz M. Management of diabetes mellitus in individuals with chronic kidney disease: therapeutic perspectives and glycemic control. *Clinics* 2016; 71: 47-53. DOI: 10.6061/clinics/2016(01)08. | Did not meet inclusion criteria |
| Bishu KG, Jenkins C, Yebyo HG, et al. Diabetes in Ethiopia: A systematic review of prevalence, risk factors, complications, and cost. Obesity Medicine 2019; 15. DOI: 10.1016/j.obmed.2019.100132 | Did not meet inclusion criteria |
| Blanco-Guillot F, Delgado-Sanchez G, Mongua-Rodriguez N, Cruz-Hervert P, Ferreyra-Reyes L, Ferreira-Guerrero E, et al. Molecular clustering of patients with diabetes and pulmonary tuberculosis: A systematic review and meta-analysis. *PLoS One* 2017; 12: e0184675. DOI: 10.1371/journal.pone.0184675. | Did not meet inclusion criteria |
| Boutayeb A, Derouich M, Boutayeb W, et al. Cerebrovascular Diseases and Associated Risk Factors in WHO Eastern Mediterranean Countries. 2014. | Did not meet inclusion criteria |
| Bukhary ZA. Rediscovering the Association Between Tuberculosis and Diabetes Mellitus: A Perspective. *Journal of Taibah University Medical Sciences* 2008; 3: 1-6. DOI: 10.1016/s1658-3612(08)70038-6. | Not systematic review |
| Chen S, Zhang Q, Dai G, Hu J, Zhu C, Su L, et al. Association of depression with pre-diabetes, undiagnosed diabetes, and previously diagnosed diabetes: a meta-analysis. *Endocrine* 2016; 53: 35-46. DOI: 10.1007/s12020-016-0869-x. | Overlapping primary studies |
| Cheng J, Zhang H, Zhao YL, Wang LX and Chen MT. Mutual Impact of Diabetes Mellitus and Tuberculosis in China. *Biomed Environ Sci* 2017; 30: 384-389. DOI: 10.3967/bes2017.051. | Did not meet inclusion criteria |
| Chin-Hong PV. Infections in Patients with Diabetes Mellitus: Importance of Early Recognition, Treatment, and Prevention. *Johns Hopkins Advanced Studies in Medicine* 2006; 6: 71-81. | Did not meet inclusion criteria |
| Chowdhury S. Depression and diabetes: a risky comorbidity. Journal of the Indian Medical Association 2004; 102: 554-556. | Not systematic review |
| Chukwuonye, II, Ogah OS, Anyabolu EN, et al. Prevalence of chronic kidney disease in Nigeria: systematic review of population-based studies. International Journal of Nephrology & Renovascular Disease 2018; 11: 165-172. Review. DOI: https://dx.doi.org/10.2147/IJNRD.S162230. | Did not meet inclusion criteria |
| Cillóniz C, Liapikou A, Ceccato A and Torres A. Risk factors for community-acquired pneumonia in adults. *Minerva Pneumologica* 2017; 56: 206-216. DOI: 10.23736/S0026-4954.17.01797-7. | HICs |
| Coleman BL, Fadel SA, Fitzpatrick T and Thomas SM. Risk factors for serious outcomes associated with influenza illness in high- versus low- and middle-income countries: Systematic literature review and meta-analysis. *Influenza Other Respir Viruses* 2018; 12: 22-29. DOI: 10.1111/irv.12504. | Did not meet inclusion criteria |
| Dalzochio Ts, Bonho L, Feksa LR and Berlese DB. Relationship between depression and diabetes Mellitus. *Revista de Ciências Médicas* 2014; 23: 91-99. | Diabetes as outcome |
| de Oliveira Junior WV, de Paula Sabino A, Figueiredo RC and Rios DR. Inflammation and poor response to treatment with erythropoietin in chronic kidney disease. *J Bras Nefrol* 2015; 37: 255-263. DOI: 10.5935/0101-2800.20150039. | Did not meet inclusion criteria |
| Deng C, Wang X and Liao Y. Current recommendations on managing tuberculosis patients with diabetes & its epidemiology. *Microb Pathog* 2016; 92: 43-45. DOI: 10.1016/j.micpath.2015.12.005. | Not systematic review |
| El-Hajj M, Salameh P, Rachidi S, et al. The epidemiology of stroke in the Middle East. European Stroke Journal 2016; 1: 180-198. Review. DOI: 10.1177/2396987316654338. | Did not meet inclusion criteria |
| The Emerging Risk Factors C. Diabetes mellitus, fasting blood glucose concentration, and risk of vascular disease: a collaborative meta-analysis of 102 prospective studies. The Lancet 2010; 375: 2215-2222. DOI: 10.1016/s0140-6736(10)60484-9. | HICs |
| Fajardo-Ortíz G. Prevalencias, discapacidades, fallecimientos y costos de la enfermedad vasculocerebral en México. Arch neurociencias 2000: 205-210. | Not systematic review |
| Feng A, Peña Y and Li W. La cardiopatía isquémica en pacientes diabéticos y no diabéticos. Rev habanera cienc méd 2017; 16: 217-228. | Not systematic review |
| Fisher-Hoch SP, Mathews CE and McCormick JB. Obesity, diabetes and pneumonia: the menacing interface of non-communicable and infectious diseases. *Trop Med Int Health* 2013; 18: 1510-1519. DOI: 10.1111/tmi.12206. | Not systematic review |

| | |
|---|---|
| Fráguas Rr, Soares SMdSR and Bronstein MD. Depressão e diabetes mellitus. *Revista de Psiquiatria Clínica* 2009; 36: 93-99. | Not systematic review |
| Gaui EN and Oliveira CCd. Doença coronária em situações especiais. Rev SOCERJ 1999; 12: 649-654. | Did not meet inclusion criteria |
| Gebre MW. Diabetes mellitus and associated diseases from Ethiopian perspective: Systematic review. Ethiopian Journal of Health Development 2013; 27: 249-253. | Did not meet inclusion criteria |
| Gonçalves Gama GG, Mussi FC and Guimarães AC. Revisando os fatores de risco cardiovascular: [revisão]. Rev enferm UERJ 2010; 18: 650-655. | Did not meet inclusion criteria |
| Gong Z and Zhao D. Cardiovascular diseases and risk factors among Chinese immigrants. Internal and emergency medicine 2016; 11: 307-318. Review. DOI: 10.1007/s11739-015-1305-6. | HICs |
| Guo C, Zhou Z, Wen Z, Liu Y, Zeng C, Xiao D, et al. Global Epidemiology of Dengue Outbreaks in 1990-2015: A Systematic Review and Meta-Analysis. *Front Cell Infect Microbiol* 2017; 7: 317. DOI: 10.3389/fcimb.2017.00317. | Did not meet inclusion criteria |
| Han X, Wang Q, Wang Y, Cai J, Ma Y, Zhou X, et al. The impact of diabetes on tuberculosis treatment outcomes: evidence based on a cumulative meta-analysis. *International Journal of Diabetes in Developing Countries* 2016; 36: 490-507. DOI: 10.1007/s13410-016-0514-5. | Did not meet inclusion criteria |
| Hayashi S and Chandramohan D. Risk of active tuberculosis among people with diabetes mellitus: systematic review and meta-analysis. Tropical Medicine and International Health 2018; 23: 1058-1070. 2018/08/01. DOI: 10.1111/tmi.13133. | Overlapping primary studies |
| Huang CQ, Dong BR, Lu ZC, Yue JR and Liu QX. Chronic diseases and risk for depression in old age: a meta-analysis of published literature. *Ageing Res Rev* 2010; 9: 131-141. DOI: 10.1016/j.arr.2009.05.005. | HICs |
| Huangfu P, Pearson F, Ugarte-Gil C and Critchley J. Diabetes and poor tuberculosis treatment outcomes: issues and implications in data interpretation and analysis. *Int J Tuberc Lung Dis* 2017; 21: 1214-1219. DOI: 10.5588/ijtld.17.0211. | Did not meet inclusion criteria |
| Huangfu P, Ugarte-Gil C, Golub J, et al. The effects of diabetes on tuberculosis treatment outcomes: an updated systematic review and meta-analysis. Int J Tuberc Lung Dis 2019; 23: 783-796. 2019/08/24. DOI: 10.5588/ijtld.18.0433. | Did not meet inclusion criteria |
| Jeon CY and Murray MB. Diabetes Mellitus Increases the Risk of Active Tuberculosis: A Systematic Review of 13 Observational Studies. *PLoS Medicine* 2008; 5: e152. DOI: 10.1371/journal.pmed.0050152. | Overlapping primary studies |
| Kalra G, Gill S, Tang TS. Depression and Diabetes Distress in South Asian Adults Living in Low- and Middle-Income Countries: A Scoping Review. Canadian Journal of Diabetes. 2020;44:521-9.e1. DOI: 10.1016/j.jcjd.2020.06.007 | Not systematic review |
| Kaze AD, Ilori T, Jaar BG, et al. Burden of chronic kidney disease on the African continent: A systematic review and meta-analysis. BMC Nephrology 2018; 19 (1) (no pagination). Review. DOI: 10.1186/s12882-018-0930-5. | Did not meet inclusion criteria |
| Kengne AP, Amoah AGB and Mbanya JC. Cardiovascular complications of diabetes mellitus in sub-Saharan Africa. Circulation 2005; 112: 3592-3601. Review. DOI: 10.1161/CIRCULATIONAHA.105.544312. | Not systematic review |
| Kengne AP, Ntyintyane LM and Mayosi BM. A systematic overview of prospective cohort studies of cardiovascular disease in sub-Saharan Africa. Cardiovascular Journal of Africa 2012; 23: 103-112. DOI: 10.5830/CVJA-2011-042. | Did not meet inclusion criteria |
| Koye DN, Magliano DJ, Nelson RG and Pavkov ME. The Global Epidemiology of Diabetes and Kidney Disease. *Adv Chronic Kidney Dis* 2018; 25: 121-132. DOI: 10.1053/j.ackd.2017.10.011. | Not systematic review |
| Lee PH, Fu H, Lee MR, et al. Tuberculosis and diabetes in low and moderate tuberculosis incidence countries. International Journal of Tuberculosis and Lung Disease 2018; 22: 7-16. DOI: 10.5588/ijtld.17.0329. | Did not meet inclusion criteria |
| Liu Q, Li W, Xue M, et al. Diabetes mellitus and the risk of multidrug resistant tuberculosis: a meta-analysis. *Scientific Reports* 2017; 7. DOI: 10.1038/s41598-017-01213-5. | Overlapping primary studies |
| Louzada SM and Vargas CR. Encefalopatia Diabética e Depressão: Dano Oxidativo No Cérebro. *Clinical & Biomedical Research* 2015; 35: 184-195. DOI: 10.4322/2357-9730.59313. | Did not meet inclusion criteria |
| Lu FP, Lin KP and Kuo HK. Diabetes and the risk of multi-system aging phenotypes: A systematic review and meta-analysis. PLoS ONE 2009; 4 (1) (no pagination). Review. DOI: 10.1371/journal.pone.0004144. | HICs |
| Mbanya JC and Sobngwi E. Diabetes microvascular and macrovascular disease in Africa. Journal of Cardiovascular Risk 2003; 10: 97-102. DOI: 10.1097/00043798-200304000-00004. | Not systematic review |
| Mezuk B, Eaton WW, Albrecht S and Golden SH. Depression and type 2 diabetes over the lifespan: a meta-analysis. *Diabetes Care* 2008; 31: 2383-2390. DOI: 10.2337/dc08-0985. | HICs |
| Miranda SS. Traramento da Tuberculose em Situações Especiais. *Pulmão RJ* 2012; 21: 68-71. | Did not meet inclusion criteria |

| | |
|---|---|
| Mohseni J, Kazemi T, Maleki MH, et al. A Systematic Review on the Prevalence of Acute Myocardial Infarction in Iran. Heart Views 2017; 18: 125-132. Review. DOI: https://dx.doi.org/10.4103/HEARTVIEWS.HEARTVIEWS_71_17. | Did not meet inclusion criteria |
| Moradi Y, Baradaran HR, Djalalinia S, et al. Complications of type 2 diabetes in Iranian population: An updated systematic review and meta-analysis. Diabetes Metab Syndr 2019; 13: 2300-2312. 2019/06/27. DOI: 10.1016/j.dsx.2019.05.018. | Did not meet inclusion criteria |
| Moreira RO, Papelbaum M, Appolinario JC, Matos AlG, Coutinho WF, Meirelles RMR, et al. Diabetes Mellitus e Depressão: Uma Revisão Sistemática. *Arquivos Brasileiros de Endocrinologia & Metabologia* 2003; 47: 19-29. | HICs |
| Nag T and Ghosh A. Cardiovascular disease risk factors in Asian Indian population: A systematic review. Journal of Cardiovascular Disease Research 2013; 4: 222-228. Review. DOI: 10.1016/j.jcdr.2014.01.004. | Did not meet inclusion criteria |
| Namale G, Kamacooko O, Kinengyere A, et al. Risk factors for hemorrhagic and ischemic stroke in Sub-Saharan Africa - A systematic review. Cerebrovascular Diseases 2018; 45 (Supplement 1): 345. Conference Abstract. DOI: 10.1159/000490132. | Abstract |
| Nantha YS. Influence of Diabetes Mellitus and Risk Factors in Activating Latent Tuberculosis Infection- A Case for Targeted Screening in Malaysia. *Medical Journal of Malaysia* 2012; 67: 467-472. | Did not meet inclusion criteria |
| Nantha YS. A Review of Tuberculosis Research in Malaysia. *Medical Journal of Malaysia* 2014; 69: 88-102. | Did not meet inclusion criteria |
| Naskar S, Victor R and Nath K. Depression in diabetes mellitus - A comprehensive systematic review of literature from an Indian perspective. *Asian Journal of Psychiatry* 2017; 27: 85-100. DOI: 10.1016/j.ajp.2017.02.018. | Diabetes as outcome |
| Noubiap JJ, Naidoo J and Kengne AP. Diabetic nephropathy in Africa: A systematic review. *World Journal of Diabetes* 2015; 6: 759-773. DOI: 10.4239/wjd.v6.i5.759. | Did not meet inclusion criteria |
| Nouwen A, Winkley K, Twisk J, Lloyd CE, Peyrot M, Ismail K, et al. Type 2 diabetes mellitus as a risk factor for the onset of depression: a systematic review and meta-analysis. *Diabetologia* 2010; 53: 2480-2486. DOI: 10.1007/s00125-010-1874-x. | HICs |
| Onen CL. Epidemiology of ischaemic heart disease in sub-Saharan Africa. Cardiovascular Journal of Africa 2013; 24: 34-42. | Did not meet inclusion criteria |
| Patel V. A short review on the association between depression and diabetes. *International Journal of Medical Science and Public Health* 2014; 3. DOI: 10.5455/ijmsph.2013.141120132. | Not systematic review |
| Patra J, Jha P, Rehm J and Suraweera W. Tobacco smoking, alcohol drinking, diabetes, low body mass index and the risk of self-reported symptoms of active tuberculosis: individual participant data (IPD) meta-analyses of 72,684 individuals in 14 high tuberculosis burden countries. *PLoS One* 2014; 9: e96433. DOI: 10.1371/journal.pone.0096433. | Did not meet inclusion criteria |
| Peters SAE, Huxley RR and Woodward M. Diabetes as a risk factor for stroke in women compared with men: a systematic review and meta-analysis of 64 cohorts, including 775385 individuals and 12539 strokes. Lancet (British edition) 2014; 383: 1973-1980. DOI: 10.1016/S0140-6736(14)60040-4. | Did not meet inclusion criteria |
| Ponte CMoM, Gurgel MHC, Ponte GAao, Ramos AVrAao and Júnior RMeM. Distúrbios metabólicos em doenças infecciosas emergentes e negligenciadas. *Arquivos Brasileiros de Endocrinologia & Metabologia* 2010; 54: 785-792. | Did not meet inclusion criteria |
| Rajendran M, Zaki RA, Aghamohammadi N. Contributing risk factors towards the prevalence of multidrug-resistant tuberculosis in Malaysia: A systematic review. Tuberculosis (Edinb). 2020;122:101925. DOI: 10.1016/j.tube.2020.101925 | Did not meet inclusion criteria |
| Rodrigues TC, Almeida FK, Ricardo ED, Biavatti K and Gamboa ML. Infecções no paciente com diabetes melito. *Rev HCPA* 2010; 30: 391-399. | Not systematic review |
| Silva DR, Muñoz-Torrico M, Duarte R, et al. Risk factors for tuberculosis: diabetes, smoking, alcohol use, and the use of other drugs. J Bras Pneumol 2018; 44: 145-152. 2018/05/24. DOI: 10.1590/s1806-37562017000000443. | Not systematic review |
| Rotella F and Mannucci E. Diabetes mellitus as a risk factor for depression. A meta-analysis of longitudinal studies. *Diabetes Res Clin Pract* 2013; 99: 98-104. 2012/12/26. DOI: 10.1016/j.diabres.2012.11.022. | HICs |
| Roy T and Lloyd CE. Epidemiology of depression and diabetes: A systematic review. *Journal of Affective Disorders* 2012; 142: S8-S21. DOI: 10.1016/S0165-0327(12)70004-6. | HICs |
| Sharma P, Visnegarwala F and Tripathi V. Burgeoning double burden of tuberculosis and diabetes in India: Magnitude of the problem – Strategies and solutions. *Clinical Epidemiology and Global Health* 2014; 2: 107-116. DOI: 10.1016/j.cegh.2013.03.002. | Did not meet inclusion criteria |
| Shen Y, Cai R, Sun J, et al. Diabetes and risk of incident chronic kidney disease in women compared with men: A systematic review and meta-analysis. Diabetologia 2016; 59 (1 Supplement 1): S490-S491. Conference Abstract. DOI: 10.1007/s00125-016-4046-9. | Abstract |
| Shen Y, Cai R, Sun J, Dong X, Huang R, Tian S, et al. Diabetes mellitus as a risk factor for incident chronic kidney disease and end-stage renal disease in women compared with | HICs |

| | |
|---|---|
| men: a systematic review and meta-analysis. *Endocrine* 2017; 55: 66-76. DOI: 10.1007/s12020-016-1014-6. | |
| Sousa GGS, Pascoal LM, Ferreira AGN, Rolim ILTP, Santos LFS, Santos Neto M. Caracterização clínico-epidemiológica da comorbidade tuberculose/diabetes mellitus: revisão integrativa. Rev enferm UERJ. 2020;28:e50255-e. DOI: 10.12957.reuerj.2020.50255 | Did not meet inclusion criteria |
| Stanifer JW, Jing B, Tolan S, Helmke N, Mukerjee R, Naicker S, et al. The epidemiology of chronic kidney disease in sub-Saharan Africa: a systematic review and meta-analysis. *The Lancet Global Health* 2014; 2: e174-e181. DOI: 10.1016/s2214-109x(14)70002-6. | Did not meet inclusion criteria |
| Stoffel C, Lorenz R, Arce M, Rico M, Fernández L and Imaz MS. Tratamiento de la tuberculosis pulmonar en un área urbana de baja prevalencia. Cumplimiento y negativización bacteriológica. *Medicina (Buenos Aires)* 2014; 74. | Did not meet inclusion criteria |
| Sumaili EK, Krzesinski JM, Cohen EP and Nseka NM. Epidemiology of chronic kidney disease in the Democratic Republic of Congo: review of cross-sectional studies from Kinshasa, the capital. *Nephrol Ther* 2010; 6: 232-239. DOI: 10.1016/j.nephro.2010.03.008. | Language (French) |
| Toledo J, George L, Martinez E, Lazaro A, Han WW, Coelho GE, et al. Relevance of Non-communicable Comorbidities for the Development of the Severe Forms of Dengue: A Systematic Literature Review. *PLoS Negl Trop Dis* 2016; 10: e0004284. DOI: 10.1371/journal.pntd.0004284. | Did not meet inclusion criteria |
| Tong A, Wang X, Li F, et al. Risk of depressive symptoms associated with impaired glucose metabolism, newly diagnosed diabetes, and previously diagnosed diabetes: a meta-analysis of prospective cohort studies. Acta Diabetologica 2016; 53: 589-598. DOI: 10.1007/s00592-016-0845-1. | HICs |
| Tsai CF, Anderson N, Thomas B, et al. Risk factors for ischemic stroke and its subtypes in Chinese vs. Caucasians: Systematic review and meta-analysis. International Journal of Stroke 2015; 10: 485-493. DOI: 10.1111/ijs.12508. | Did not meet inclusion criteria |
| Tsai CF, Anderson N, Thomas B, et al. Comparing risk factor profiles between intracerebral hemorrhage and ischemic stroke in Chinese and white populations: Systematic review and meta-analysis. PLoS ONE 2016; 11 (3) (no pagination). Review. DOI: 10.1371/journal.pone.0151743. | Did not meet inclusion criteria |
| Tsai WC, Wu HY, Peng YS, Ko MJ, Wu MS, Hung KY, et al. Risk Factors for Development and Progression of Chronic Kidney Disease: A Systematic Review and Exploratory Meta-Analysis. *Medicine (Baltimore)* 2016; 95: e3013. DOI: 10.1097/MD.0000000000003013. | HICs |
| Ugarte-Gil C and Moore DA. Comorbilidad de Tuberculosis y Diabetes: Problema Aún Sin Resolver. *Revista Peruana de Medicina Experimental y Salud Pública* 2014; 31: 137-142. | Did not meet inclusion criteria |
| Vardakas KZ, Siempos, II and Falagas ME. Diabetes mellitus as a risk factor for nosocomial pneumonia and associated mortality. Diabet Med 2007; 24: 1168-1171. 2007/09/25. DOI: 10.1111/j.1464-5491.2007.02234.x. | Not systematic review |
| Villasis-Keever MA, Rendon-Masias ME, Pineda-Cruz R, et al. A meta-analysis of cardiovascular risk factors: Which is the difference between men and women? Value in Health 2010; 13 (3): A151. Conference Abstract. DOI: 10.1016/S1098-3015%2810%2972731-5. | Abstract |
| Wagner FA, González-Forteza C, Sánchez-García S, García-Peña C and Gallo JJ. Enfocando la depresión como problema de salud pública en México. *Salud Mental* 2012; 35: 3-11. | Did not meet inclusion criteria |
| Wagnew F, Eshetie S, Alebel A, et al. Meta-analysis of the prevalence of tuberculosis in diabetic patients and its association with cigarette smoking in African and Asian countries. BMC Res Notes 2018; 11: 298. DOI: https://dx.doi.org/10.1186/s13104-018-3390-x. | Did not meet inclusion criteria |
| Wang J, Wen X, Li W, et al. Risk Factors for Stroke in the Chinese Population: A Systematic Review and Meta-Analysis. Journal of Stroke and Cerebrovascular Diseases 2017; 26: 509-517. DOI: 10.1016/j.jstrokecerebrovasdis.2016.12.002. | Did not meet inclusion criteria |
| Wang H, Ba Y, Cai RC, et al. Association between diabetes mellitus and the risk for major cardiovascular outcomes and all-cause mortality in women compared with men: a meta-analysis of prospective cohort studies. BMJ Open 2019; 9: e024935. 2019/07/20. DOI: 10.1136/bmjopen-2018-024935. | Did not meet inclusion criteria |
| Workneh MH, Bjune GA and Yimer SA. Prevalence and associated factors of tuberculosis and diabetes mellitus comorbidity: A systematic review. *PLoS One* 2017; 12: e0175925. DOI: 10.1371/journal.pone.0175925. | Did not meet inclusion criteria |
| Wu HY, Peng YS, Wu MS, et al. Risk factors of developmentand progression of chronic kidney disease: A systematic reviewand meta-analysis. Nephrology Dialysis Transplantation 2014; 3): iii126-iii127. Conference Abstract. DOI: 10.1093/ndt/gfu146. | Abstract |
| Zhuang Q-S, Shen L and Ji H-F. Quantitative assessment of the bidirectional relationships between diabetes and depression. *Oncotarget* 2017; 8: 23389-23400. | HICs |

## Excluded after quality assessment (n=9)

| **Reference** | **Reason for Exclusion** |
|---|---|
| Amini M and Parvaresh E. Prevalence of macro- and microvascular complications among patients with type 2 diabetes in Iran: A systematic review. Diabetes Research and Clinical Practice 2009; 83: 18-25. Review. DOI: 10.1016/j.diabres.2008.10.010. | Critically low confidence level |
| Htun NS, Odermatt P, Eze IC, et al. Is Diabetes a Risk Factor for a Severe Clinical Presentation of Dengue? - Review and Meta-analysis. *PLoS Neglected Tropical Diseases* 2015; 9: e0003741. DOI: 10.1371/journal.pntd.0003741. | Critically low confidence level |
| Kazeminia M, Salari N, Mohammadi M. Prevalence of Cardiovascular Disease in Patients with Type 2 Diabetes Mellitus in Iran: A Systematic Review and Meta-Analysis. Journal of Diabetes Research. 2020;2020 (no pagination). DOI: 10.1155/2020/3069867 | Critically low confidence level |
| Khaledi M, Haghighatdoost F, Feizi A, et al. The prevalence of comorbid depression in patients with type 2 diabetes: an updated systematic review and meta-analysis on huge number of observational studies. *Acta Diabetol* 2019; 56: 631-650. 2019/03/25. DOI: 10.1007/s00592-019-01295-9. | Critically low confidence level |
| Lopes A, Perry IS, Bavaresco DV, et al. Association between Major Depression and Type 2 Diabetes Mellitus: a Meta-Analysis and Meta-Regression of Observational Studies. *InternatIonal archIves of MedIcIne, sectIon: PsychIatry & Mental health* 2016; 9. DOI: 10.3823/1920. | Critically low confidence level |
| Pashaki MS, Mezel JA, Mokhtari Z, Gheshlagh RG, Hesabi PS, Nematifard T, et al. The prevalence of comorbid depression in patients with diabetes: A meta-analysis of observational studies. Diabetes Metab Syndr. 2019;13:3113-9. DOI: 10.1016/j.dsx.2019.11.003 | Critically low confidence level |
| Teshome HM, Ayalew GD, Shiferaw FW, et al. The Prevalence of Depression among Diabetic Patients in Ethiopia: A Systematic Review and Meta-Analysis, 2018. Depress Res Treat 2018; 2018: 6135460. 2018/06/29. DOI: 10.1155/2018/6135460. | Critically low confidence level |
| Windhy M, Vitri W, Endang Sutisna S. Meta-analysis on the effects of hypertension, type ii diabetes mellitus, and smoking on the risk of stroke among Asian young adult population. Journal of Epidemiology and Public Health. 2019;4:296-306. DOI: 10.26911/jepublichealth.2019.04.04.04 | Critically low confidence level |
| Yong H, Foody J, Linong J, et al. A Systematic Literature Review of Risk Factors for Stroke in China. Cardiology in Review 2013; 21: 77-93. DOI: 10.1097/crd.0b013e3182748d37. | Critically low confidence level |

## Table S4: Quality assessment results using adapted AMSTAR 2 checklist

| Author, Year | Outcome | Q1. PICO components | Q2. Written protocol | Q3. Study design inlusion rationale | ***Q4. Comprehensive search strategy** | Q5. Duplicate study selection | Q6. Duplicate data extraction | Q7. Excluded studies list | ***Q8. Sufficient study details** | ***^Q9. RoB assessment** | ***^Q10. Quality appraisal** | Q11. Study funding | ***Q12. Appropriate M-A method** | Q13. RoB impact on M-A | ***Q14. RoB impact on results** | Q15. Heterogeneity in results | ***Q16. Publication bias and impact** | Q17. Conflict of interest reporting | Confidence |
|---|---|---|---|---|---|---|---|---|---|---|---|---|---|---|---|---|---|---|---|
| Einarson, 2018 | CVD | Y | N | Y | PY | Y | Y | N | PY | PY | PY | Y | - | - | Y | Y | - | Y | Moderate |
| Poorzand, 2019 | CVD | Y | N | N | PY | Y | Y | N | PY | PY | PY | N | Y | Y | N | Y | Y | Y | Low |
| Koye, 2017 | CKD | Y | N | Y | PY | Y | N | N | Y | PY | Y | N | - | - | Y | Y | - | Y | Moderate |
| Shiferaw, 2020 | CKD | Y | Y | Y | PY | Y | Y | N | PY | Y | Y | N | Y | Y | N | Y | Y | Y | Low |
| Mendenhall, 2014 | Depression | Y | N | N | PY | Y | N | N | PY | N | N | N | - | - | Y | Y | - | Y | Low |
| Uphoff, 2019 | Depression | Y | Y | Y | PY | Y | Y | N | N | Y | Y | Y | Y | Y | Y | Y | Y | Y | Moderate |
| Hussain, 2018 | Depression | Y | PY | Y | PY | Y | Y | N | Y | PY | Y | N | Y | Y | Y | Y | Y | Y | Moderate |
| Khalighi, 2019 | Depression | Y | N | N | PY | Y | Y | N | PY | PY | PY | N | Y | Y | Y | Y | Y | Y | Moderate |
| Wang, 2019 | Depression | Y | N | Y | PY | Y | Y | N | PY | PY | Y | N | Y | Y | Y | Y | Y | Y | Moderate |
| Bailey & Ayles, 2017 | TB | Y | N | Y | PY | N | N | N | Y | PY | Y | N | - | - | Y | Y | - | Y | Moderate |
| McMurry, 2018 | TB | Y | N | Y | PY | Y | N | N | PY | Y | Y | N | - | - | Y | Y | - | Y | Moderate |
| Al-Rifai, 2017 | TB | Y | N | Y | PY | N | Y | N | Y | PY | PY | N | Y | Y | Y | Y | Y | Y | Moderate |
| Lee, 2017 | TB | Y | N | Y | PY | Y | N | N | PY | PY | Y | N | Y | Y | Y | Y | Y | Y | Moderate |
| Tegegne, 2018 | TB | Y | Y | Y | PY | Y | Y | Y | PY | Y | Y | N | Y | Y | Y | Y | Y | Y | High |
| **Excluded due to critically low confidence level** | | | | | | | | | | | | | | | | | | | |
| Khaledi, 2019 | Depression | Y | N | Y | PY | Y | Y | N | N | N | N | N | Y | Y | Y | Y | Y | Y | Critically Low |
| Lopes, 2016 | Depression | Y | N | N | PY | Y | N | N | N | N | N | N | Y | Y | N | Y | Y | N | Critically Low |
| Pashaki, 2019 | Depression | Y | N | Y | PY | Y | Y | N | N | Y | PY | N | Y | Y | N | Y | Y | Y | Critically Low |

| | | | | | | | | | | | | | | | | | | | |
|---|---|---|---|---|---|---|---|---|---|---|---|---|---|---|---|---|---|---|---|
| Teshome, 2018 | Depression | Y | N | Y | **PY** | Y | Y | N | **N** | **Y** | **PY** | N | **N** | N | **N** | Y | **Y** | Y | **Critically Low** |
| Amini, 2009 | CVD | Y | N | Y | N | N | N | N | **N** | **N** | **N** | N | **-** | **-** | **N** | N | **-** | Y | **Critically Low** |
| Kazeminia, 2019 | CVD | Y | N | Y | **PY** | Y | N | N | **N** | **Y** | **Y** | N | **Y** | Y | **N** | N | **Y** | Y | **Critically Low** |
| Windhy, 2019 | CVD | Y | N | N | **PY** | N | N | N | **PY** | **N** | **N** | N | **Y** | N | **N** | N | Y | Y | **Critically Low** |
| Yong, 2013 | CVD | Y | N | N | **PY** | N | Y | N | **Y** | **N** | **N** | N | **-** | **-** | **N** | Y | **-** | N | **Critically Low** |
| Htun, 2015 | Dengue | Y | N | Y | **N** | N | Y | N | **Y** | **N** | **N** | N | **Y** | Y | **Y** | Y | **N** | Y | **Critically Low** |

***** Critical items - if any of these questions received a 'N,' it is automatically dropped to a low confidence level.
**^** Q9 and Q10 counted together as one critical item. If either item received a 'N,' it will count as missing one critical item rather than two.

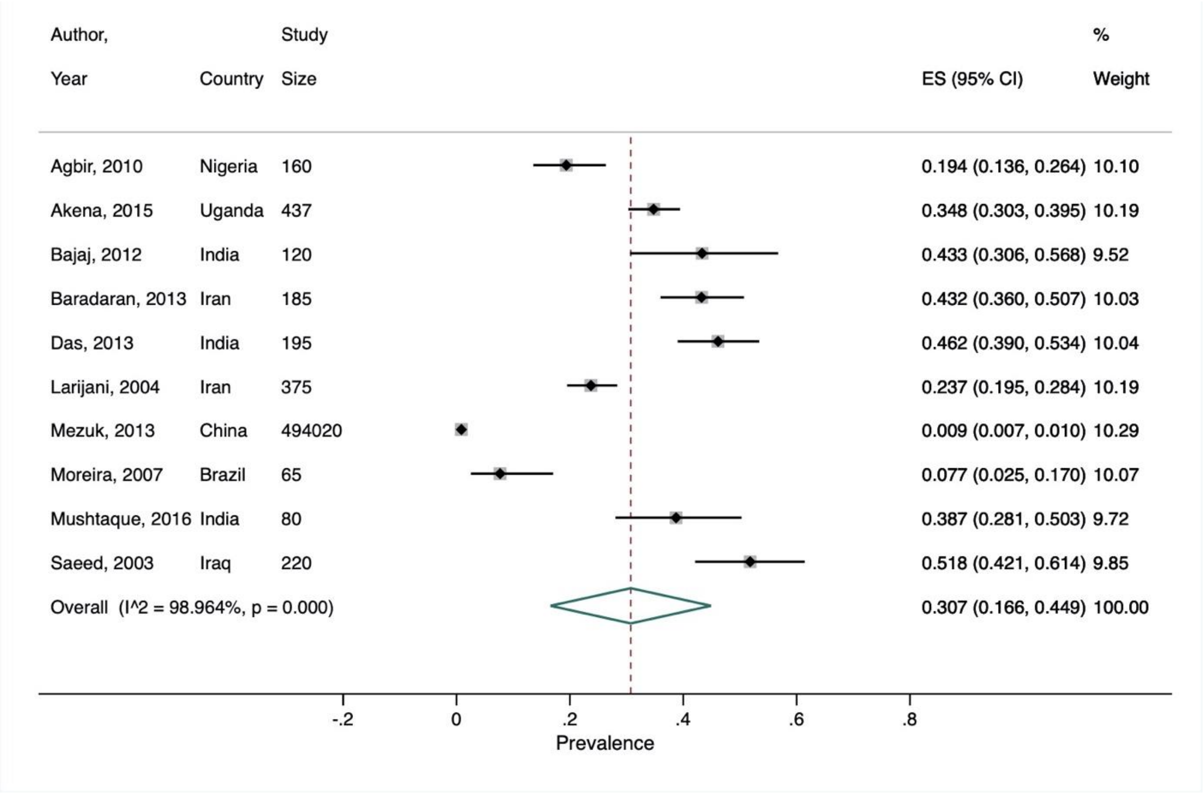


**Figure S1.** Forest plot of prevalence studies from the Wang *et al.* [32] meta-analysis on the association between diabetes and depression.

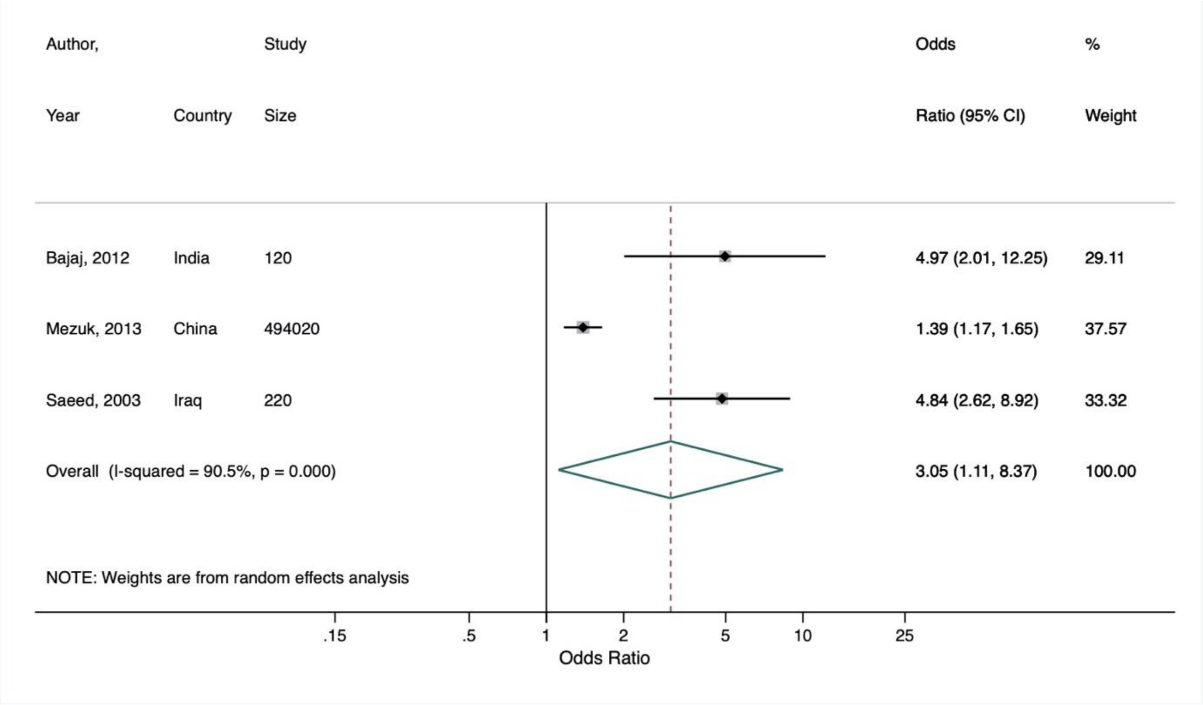


**Figure S2.** Forest plot of odds ratio studies from the Wang *et al.* [32] meta-analysis on the association between diabetes and depression.

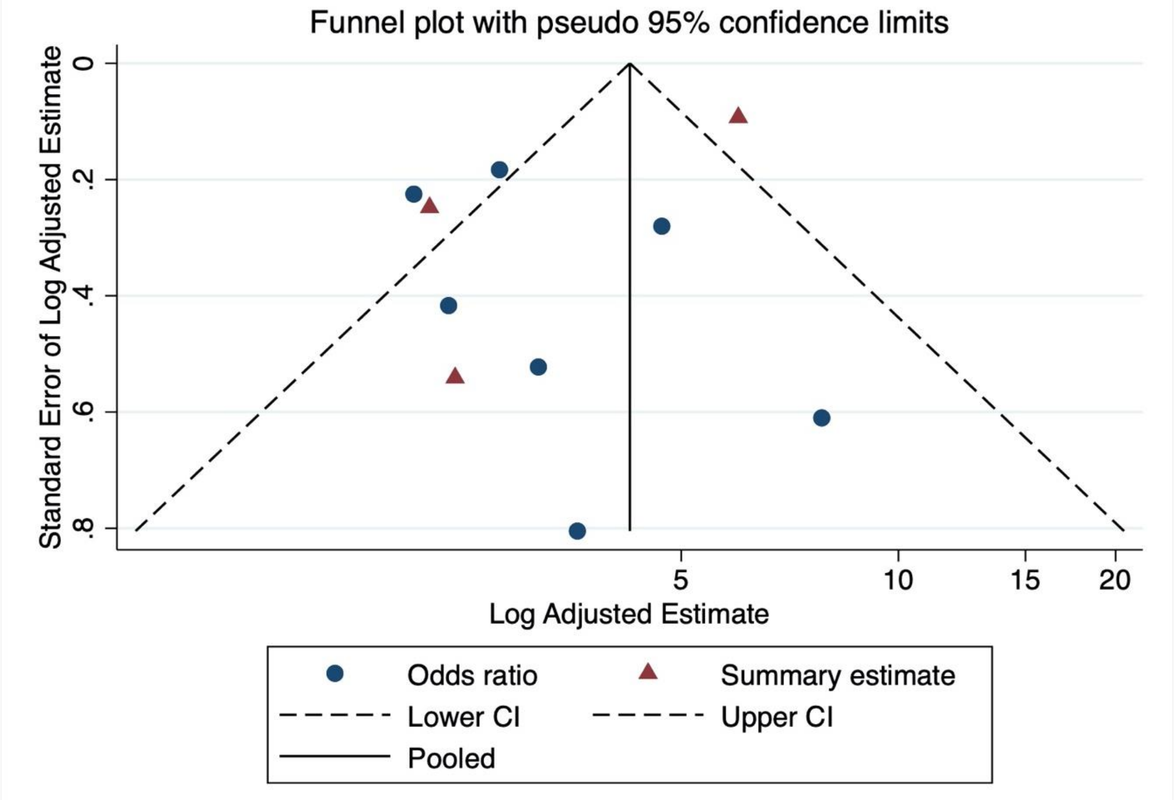


**Figure S3.** Funnel plot to test for publication bias in Al-Rifai *et al.* [35] review on the association between diabetes and tuberculosis.

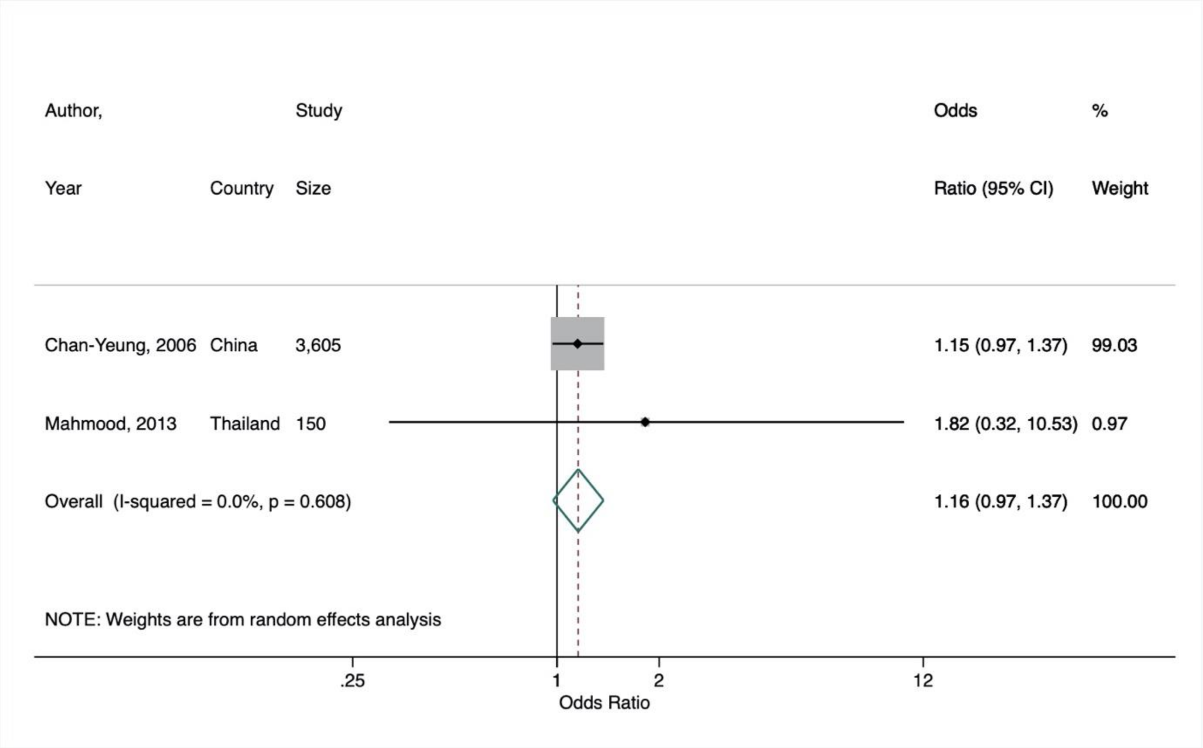


**Figure S4.** Secondary meta-analysis of studies included in Lee et al. [36] showing the study-specific and summary estimates of the odds of latent tuberculosis among people with diabetes.

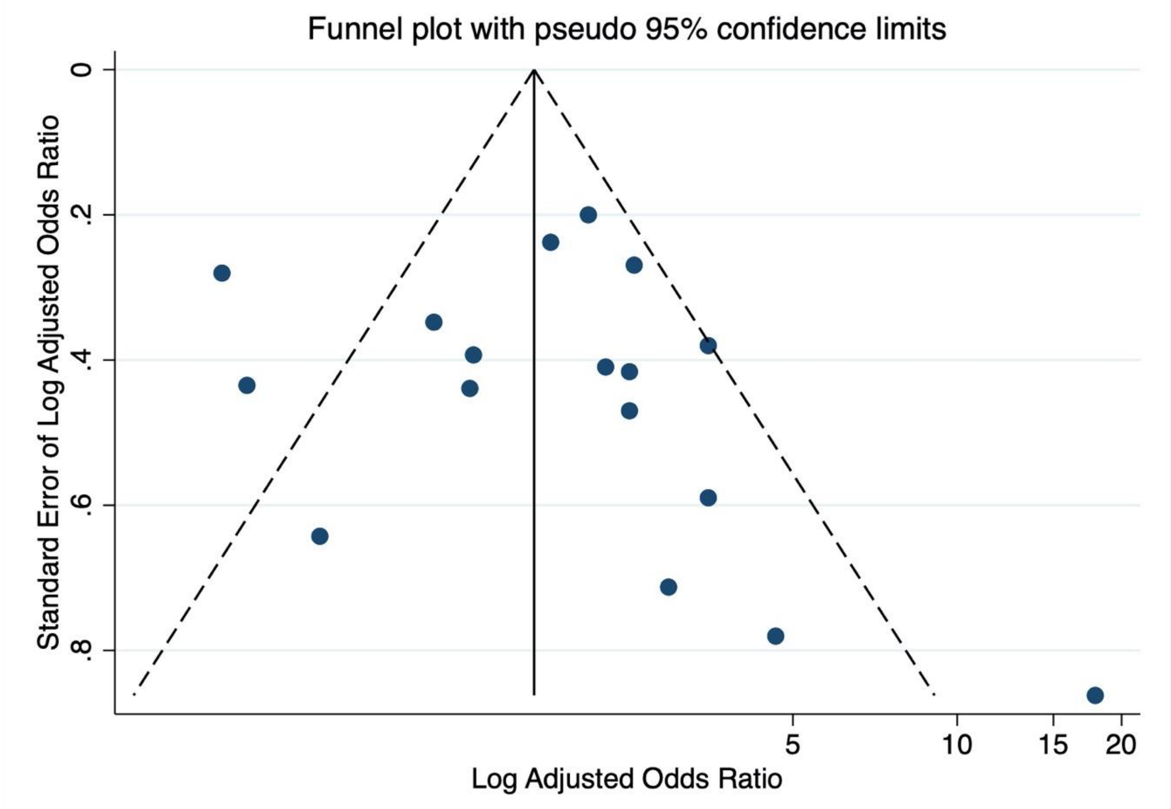


**Figure S5.** Funnel plot to test for publication bias in Tegegne *et al.* [37] review on the association between diabetes and multi-drug resistant tuberculosis.
